# Assessing the Effectiveness of eHealth Interventions to Manage Multiple Lifestyle Risk Behaviors Among Older Adults: Systematic Review and Meta-Analysis

**DOI:** 10.2196/58174

**Published:** 2024-07-31

**Authors:** Beibei Shi, Guangkai Li, Shuang Wu, Hongli Ge, Xianliang Zhang, Si Chen, Yang Pan, Qiang He

**Affiliations:** 1 School of Physical Education, Shandong University Jinan China; 2 School of Nursing and Rehabilitation, Cheeloo College of Medicine, Shandong University Jinan China

**Keywords:** eHealth, lifestyle risk behaviors, older adults, multiple health behavior change, mobile phone

## Abstract

**Background:**

Developing adverse lifestyle behaviors increases the risk of a variety of chronic age-related diseases, including cardiovascular disease, obesity, and Alzheimer disease. There is limited evidence regarding the effectiveness of eHealth-based multiple health behavior change (MHBC) interventions to manage lifestyle risk behaviors.

**Objective:**

The purpose of this systematic evaluation was to assess the effectiveness of eHealth MHBC interventions in changing ≥2 major lifestyle risk behaviors in people aged ≥50 years.

**Methods:**

The literature search was conducted in 6 electronic databases—PubMed, Embase, Web of Science, Scopus, Cochrane Library, and SPORTDiscus—from inception to May 1, 2024. Eligible studies were randomized controlled trials of eHealth interventions targeting ≥2 of 6 behaviors of interest: alcohol use, smoking, diet, physical activity (PA), sedentary behavior, and sleep.

**Results:**

A total of 34 articles with 35 studies were included. eHealth-based MHBC interventions significantly increased smoking cessation rates (odds ratio 2.09, 95% CI 1.62-2.70; *P*<.001), fruit intake (standardized mean difference [SMD] 0.18, 95% CI 0.04-0.32; *P*=.01), vegetable intake (SMD 0.17, 95% CI 0.05-0.28; *P*=.003), self-reported total PA (SMD 0.22, 95% CI 0.02-0.43; *P*=.03), and objectively measured moderate to vigorous PA (SMD 0.25, 95% CI 0.09-0.41; *P*=.002); in addition, the interventions decreased fat intake (SMD –0.23, 95% CI –0.33 to –0.13; *P*<.001). No effects were observed for alcohol use, sedentary behavior, or sleep. A sensitivity analysis was conducted to test the robustness of the pooled results. Moreover, the certainty of evidence was evaluated using the GRADE (Grading of Recommendations Assessment, Development, and Evaluation) framework.

**Conclusions:**

eHealth-based MHBC interventions may be a promising strategy to increase PA, improve diet, and reduce smoking among older adults. However, the effect sizes were small. Further high-quality, older adult–oriented research is needed to develop eHealth interventions that can change multiple behaviors.

**Trial Registration:**

PROSPERO International Prospective Register of Systematic Reviews CRD42023444418; https://www.crd.york.ac.uk/prospero/display_record.php?ID=CRD42023444418

## Introduction

### Background

The aging population and the rise in chronic diseases will significantly increase health care expenditures; in the United States, for example, it is estimated that 80% of older adults have 1 chronic disease and 50% have at least 2 [1]. Up to 80% of global cases of heart disease, stroke, and type 2 diabetes, as well as >30% of cancers, could be prevented by reducing tobacco and harmful alcohol use and improving diet and physical activity (PA) [2]. Key indicators of health risk behaviors in older adults include physical inactivity, eating <5 portions of fruits and vegetables per day, obesity, and current tobacco use. The American Heart Association’s recently published Life’s Essential 8 metrics include healthy sleep as an indicator of cardiovascular health on top of a healthy diet, participation in PA, and avoidance of nicotine [3]. Similarly, many health guidelines now integrate recommendations for PA, sleep, and sedentary behavior (SB) [4,5]. Overall, there is a clear link between these risk factors and poor health outcomes and quality of life [6,7]. Lifestyle behaviors, as important factors in health, provide scientists with valuable and interesting areas of research [8,9].

Engaging in multiple risk behaviors can negatively impact health by increasing the risk of, for example, chronic disease and mortality [10]; moreover, risk behaviors often occur simultaneously. A previous study reported a prevalence rate of approximately 50% for the co-occurrence of unhealthy diet and physical inactivity in adults [11]. Compared to those who do not engage in any of the 4 lifestyle risk behaviors, those who do so face an elevated mortality risk equal to an additional 14 years of aging in both healthy populations [12] and populations with disease [13,14]. This suggests that it may be beneficial to use a holistic intervention approach to collectively change multiple health behaviors rather than individually change a single behavior. Multiple health behavior change (MHBC) interventions have attracted increased attention of late [15,16]. Growing evidence suggests that lifestyle interventions targeting MHBC may have a greater impact on public health than interventions targeting single health behavior change (SHBC) [17,18]. The advantages of MHBC interventions include maximizing health benefits and greater reduction in medical costs [19]; in addition, successfully modifying 1 behavior may increase confidence or motivation to change other health behaviors [16]. Existing systematic reviews have examined the effectiveness of behavioral interventions on multiple health risk behaviors [20].

eHealth refers to the use of information and communication technologies in health and health-related fields to enhance health care services; health surveillance; health literature; and health education, knowledge, and research [21]. There are several advantages over traditional face-to-face interventions [22]. An eHealth intervention can be delivered over long distances; is cost-effective, efficient, and highly accessible; and allows for easy data collection [22,23]. This makes eHealth interventions potentially powerful and scalable tools and enables eHealth-based MHBC interventions to improve ongoing adherence to chronic disease management. However, only a few systematic reviews have specifically examined the effectiveness of eHealth-based MHBC interventions in adult populations; for example, the review by Norman et al [24] focused on dietary behavior change and PA interventions, while Oosterveen et al [25] examined interventions targeting smoking, nutrition, alcohol use, and PA. Overall, previous reviews have included a limited number of health behaviors (<4), focusing primarily on PA and diet.

### Objectives

Although studies on eHealth-based MHBC interventions aimed at promoting lifestyle changes in adolescents [26] and adults have been published, to the best of our knowledge, there are no knowledge reviews of such interventions designed for the older population. Older adults have limited access to programs and services that promote healthy lifestyles (eg, gyms) compared to younger adults [27]. eHealth interventions may be the solution to help individuals adopt and maintain a healthy lifestyle. Therefore, it is urgent to investigate whether eHealth can be used to implement evidence-based lifestyle changes. In this review, we targeted studies involving adults aged ≥50 years because this age is often associated with retirement, providing more time and energy to focus on health, and improved quality of life is critical for older adults. A previous systematic review found significant effects of eHealth interventions on PA in older adults; however, lifestyle risk behaviors other than PA were not part of the inclusion criteria, and the findings were inconclusive due to an insufficient number of studies [28]. Engaging in multiple lifestyle risk behaviors increases the risk of chronic disease and all-cause mortality more than the cumulative effect of a single behavior [29,30]. Therefore, we aimed to systematically review the effectiveness of eHealth interventions for risk behavior change in older adults, targeting ≥2 of 6 behaviors of interest: alcohol use, smoking, diet, PA, SB, and sleep.

## Methods

### Study Design

This systematic review and meta-analysis was registered with PROSPERO (CRD42023444418) and was reported following the PRISMA (Preferred Reporting Items for Systematic Reviews and Meta-Analyses) guidelines (refer to Multimedia Appendix 1 for the PRISMA checklist) [31]. In addition, this systematic review and meta-analysis was conducted according to the recommendations of the Cochrane Handbook for Systematic Reviews of Interventions [32].

### Search Strategy

We conducted a literature search from inception to May 1, 2024, in 6 databases: PubMed, Embase, Cochrane Library, Scopus, Web of Science, and SPORTDiscus. Following the PICOS (Population, Intervention, Comparison, Outcomes, and Study Design) principles and recommendations from the Cochrane Collaboration, we designed the search strategy using Medical Subject Headings terms, text word searches, and Boolean logic (Multimedia Appendix 2). In addition, we incorporated keywords, titles, or abstract terms, including but not limited to “older adults,” “health behavior,” “risk reduction behavior,” “ehealth,” “mobile health,” and “telemedicine.” The search was restricted to randomized controlled trials (RCTs), and the studies included were limited to those published in English. Furthermore, we manually reviewed the reference lists of the retrieved studies and identified and obtained other relevant literature.

### Study Eligibility Criteria

The study eligibility criteria are presented in Textbox 1.

**Inclusion and exclusion criteria.**

**Inclusion criteria**

- Population: mean age: ≥50 years
- Intervention: eHealth is a major component of the intervention
- Comparison: included no intervention, education as usual, or an alternative evidence-based intervention not delivered via eHealth (eg, face-to-face)
- Outcome: targeted ≥2 of the following behaviors: drinking, smoking, diet, physical activity, sedentary behavior, and sleep
- Study design: randomized controlled trials
- Language: English

**Exclusion criteria**

- Population: mean age: <50 years
- Intervention: eHealth component of the intervention is not predominant (eg, face-to-face interventions complemented by the use of a website)
- Comparison: any intervention delivered via eHealth component
- Outcome: targeted 1 of the following behaviors: drinking, smoking, diet, physical activity, sedentary behavior, and sleep
- Study design: qualitative studies, conference articles, letters, reviews, commentaries, protocols, or pilot studies
- Language: not English

### Study Selection and Data Extraction

All titles and abstracts of the identified studies were downloaded and imported into EndNote X9 (Clarivate), and all duplicate articles were removed. To confirm whether the studies met the included criteria, 2 authors (BS and GL) worked independently to screen the titles and abstracts of the studies simultaneously. Next, they reviewed the full text of each paper based on the eligibility criteria. Any disagreements were resolved through discussion or consultation with a third author (QH).

Two authors (BS and GL) extracted data independently from the included studies and entered them into a predesigned data extraction form. The data extracted included the first author, the year of publication, the country where the study took place, study design, intervention frequency and duration, participant population, age, sample size, targeted risk behaviors, measurement tools, intervention types, intervention components, types of control groups, and theoretical basis or behavior change techniques.

### Quality Assessment

Two reviewers (BS and GL) independently examined the methodological quality and risk of bias of the included studies using the risk-of-bias tool for randomized trials in the Cochrane Handbook for Systematic Review of Interventions (version 5.1.0). The tool includes 6 domains: selection bias (random sequence generation and allocation concealment), performance bias (blinding of participants and personnel), detection bias (blinding of outcome assessment), attrition bias (incomplete outcome data), reporting bias (selective reporting), and other bias (anything else, ideally prespecified). Each domain has 3 grades: low risk of bias, high risk of bias, and unclear risk of bias. Disagreements were resolved through discussion or consultation with a third author.

In addition, we used the GRADE (Grading of Recommendations Assessment, Development, and Evaluation) framework to assess the quality of the body of evidence [33].

### Data Synthesis and Analysis

For our meta-analysis, the outcomes were prevalence of alcohol use and smoking (*yes* or *no*); intake of fruit and vegetables (mean servings per day or mean portions per day), sugar intake, fat intake, fiber intake (percentage of total energy, grams per 1000 kilocalories, or grams per day), and energy intake (kilojoules per day or kilocalories per day); PA (accelerometer and self-report; minutes per day or minutes per week); sedentary time (minutes per day); and sleep (minutes per day or hours per day). For continuous outcomes, the corresponding variance was calculated using the preintervention and postintervention means and SDs; and for dichotomous outcomes, we extracted the preintervention and postintervention change values and sample sizes. However, if some studies had changes in baseline and postintervention data or if there were significant differences in their baseline data, we used the within-group difference in means and SDs for the intervention and control groups to calculate the effect size. Review Manager (version 5.3; The Cochrane Collaboration) was used to conduct the meta-analysis. We report continuous outcomes using standardized mean differences (SMDs) and dichotomous outcomes using odds ratios (ORs). We present these results using forest plots for each outcome of interest, with the weight (in percentage) indicating the influence of an individual study on the pooled result. The overall effect difference was considered statistically significant if the 2-tailed *P* value was <.05. In addition, we assessed the statistical heterogeneity of the included studies using the *I*^2^ statistic and *P* value. Data were pooled and analyzed with a fixed effects model if *I*^2^≤50% and *P*>.10; if these values were not met, which meant high heterogeneity among the studies, we used a random effects model to obtain more conservative estimates. We also conducted a sensitivity analysis using the leave-1-study-out method to test the robustness and reliability of the pooled results.

## Results

### Search Results and Study Selection

**Figure 1:**
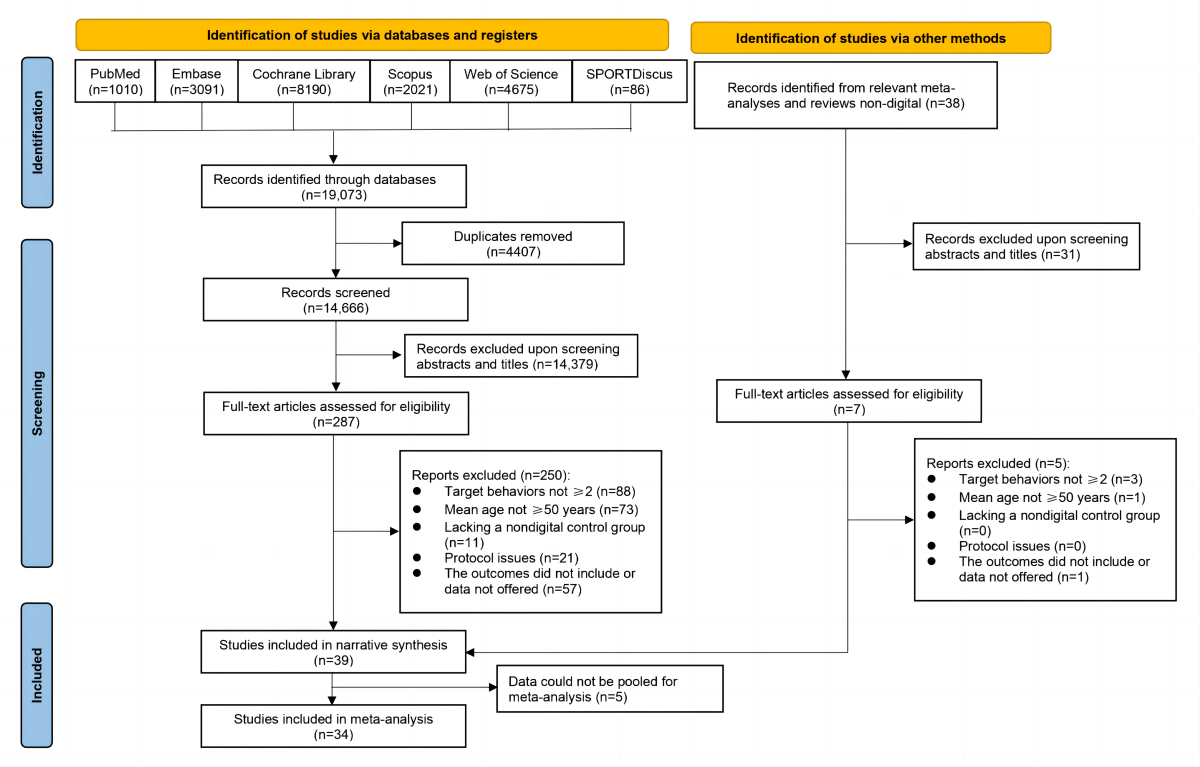
PRISMA (Preferred Reporting Items for Systematic Reviews and Meta-Analyses) flowchart showing the article selection process.

Across the 6 electronic databases, a total of 19,073 articles were initially retrieved from the literature search. Of these 19,073 articles, 4407 (23.11%) duplicates were removed. Of the remaining 14,666 articles, 14,379 (98.04%) were excluded after title and abstract screening, leaving 287 (1.96%) full-text articles. Of these 287 articles, 250 (87.1%) were excluded for reasons related to the eligibility criteria, leaving 37 (12.9%) studies for inclusion in the narrative synthesis. Using other methods, an additional 38 records were identified, of which 31 (81.6%) were excluded after title and abstract screening. Of the remaining 7 articles assessed for eligibility, 5 (71%) were excluded, leaving 2 (29%) studies for inclusion. The total number of articles included in the narrative synthesis was 39, of which 5 (13%) articles whose data could not be pooled for meta-analysis were excluded. Ultimately, 34 articles and 35 studies were included in the systematic review. The search and selection processes are shown in Figure 1.

### Description of the Included Studies

#### Study Characteristics

Table 1 presents the study characteristics of the 35 studies from the 34 included articles. All 35 studies are RCTs that were conducted between 2009 and 2024 in 15 countries: the United States (n=10, 29%), Australia (n=5, 14%), the United Kingdom (n=3, 9%), India (n=2, 6%), Canada (n=3, 9%), China (n=2, 6%), Belgium (n=2, 6%), Sweden (n=1, 3%), Germany (n=1, 3%), Italy (n=1, 3%), the Netherlands (n=1, 3%), South Korea (n=1, 3%), Israel (n=1, 3%), Japan (n=1, 3%), and Iran (n=1, 3%).

**Table 1:** Characteristics of the included studies.

Study, year; country	Population	Sample population; mean age (SD); female population (%)	Targeted risk behaviors	Study design; intervention duration; frequency	Intervention	Measurement tools	Intervention components	Comparison group	Theoretical basis or behavior change techniques
Abu-Saad et al [34], 2019; Israel	Patients with type 2 diabetes mellitus	n=50; age: mean 53.0 (SD 7.6) y; 29.58% women	PA^a^; diet	2 arms; 12 mo; personalized information service	Windows software	Questionnaires	The intervention was based on a Windows platform designed as a multifunctional tool that collected data on habitual eating and PA behaviors, personalized by identifying lifestyle behaviors that needed to be improved as targets for education and behavior change, focusing and prioritizing the education and counseling process.	Standard lifestyle advice	Behavior change techniques
Babu et al [35], 2024; India	Patients with fatty liver disease	n=209; age: mean 60.4 (SD 10.0) y; 23.4% women	PA; diet; alcohol use; smoking	2 arms; 6 mo; 29 SMS text messages and 4 videos	Telephone	Self-reporting	Participants in the intervention group were instructed to install the app, and 29 SMS text messages were sent for medication adherence, risk factor control, and lifestyle and behavior change; in addition, 4 videos on various aspects of stroke were sent to participants in the intervention group on scheduled days of the month.	Usual care	NR^b^
Bae et al [36], 2021; South Korea	Patients with coronary heart disease	n=879; age: mean 60.4 (SD 10.5) y; 83.3% women	PA; smoking	2 arms; 6 mo; 4 times/wk	SMS text messages	PA: IPAQ^c^	Messages were based on the TEXT ME^d^ trial and the Australian Heart Foundation Healthy Living Guidelines.	Usual care	NR
Bantum et al [37], 2014; United States	Survivors of cancer	n=352; age: mean 50.9 (SD 11.0) y; 82.1% women	PA; diet; sleep	2 arms; 6 mo; 6 wk	Web-based	PA: Godin Leisure Time Exercise Questionnaire; diet: Block Food Frequency Questionnaire; sleep: 5-item validated Women's Health Initiative Insomnia Rating Scale	The intervention was based on online seminars, covering topics such as diet, exercise, and stress management; participants set weekly action plans and receive facilitator feedback on their progress through a *Discussion Center* update; the intervention website includes, apart from *Discussion Center*, sections labeled *My Tools*, *Post Office*, and *Help*, promoting social interaction and personal behavior shaping in addition to providing links to resources.	Waitlist	Behavior change theory
Bloom et al [38], 2020; United States	Patients with chronic obstructive pulmonary disease	n=22; age: mean 70 (SD 7.4) y; 40.9% women	PA; smoking; alcohol use; diet	2 arms; 3 mo; NR	Social networking tool	PA: IPAQ, short-form version; diet: FFQ^e^	The intervention was based on an online social tool that provided individuals with multiple levels of self-management support; the online tool assisted users in mapping personal support networks, understanding preferences, and guiding participation in valuable social activities; the intervention was delivered by trained researchers, and participants had the option of receiving the link via email.	Usual care	NR
Campbell et al [39], 2009; United States	Patients with colon and rectal cancer	n=735; age: mean 66.5 (SD 10.0) y; 49.4% women	PA: NR; diet	4 arms; 12 mo; 4 times in total	Telephone	PA: 7-Day Physical Activity Recall questionnaire; diet: FFQ	The intervention components included using a client-centered collaborative decision-making approach, giving nonjudgmental feedback, allowing for resistance, and encouraging the participant to make the argument for change; interviewers relied on using open-ended questions and reflections to draw out participants’ motives and desires regarding behavior change.	Tailored print communication intervention	Health behavior theories, transtheoretical model (stages of change), and social cognitive theory
Chow et al [40], 2015; Australia	Patients with coronary heart disease	n=710; age: mean 57.6 (SD 9.2) y; 18% women	PA; smoking	2 arms; 6 mo; 4 times/wk	SMS text messages	PA: GPAQ^f^	The SMS text messaging–based prevention program sent participants 4 semipersonalized messages per week for 24 weeks, including advice on diet, PA, and smoking cessation; messages were selected based on an algorithm and participants’ baseline characteristics and sent randomly through a management system; participants received brief training to monitor message responses and did not respond interactively.	Usual care	NR
Cicolini et al [41], 2014; Italy	Patients with hypertension	n=198; age: mean 59.0 (SD 14.5) y; 49% women	PA; smoking; alcohol use; diet	2 arms; 6 mo; 1 time/wk	Email and telephone	Questionnaires	The intervention was based on email reminders for 6 months covering advice on diet, exercise, smoking cessation, and more; recommendations were based on current healthy lifestyle guidelines.	Usual care	NR
Djuric et al [42], 2011; United States	Women with breast cancer	n=30; age: mean 52.3 (SD 9.5) y	PA; diet	2 arms; 12 mo; NR	Telephone	PA: Women’s Health Initiative questionnaire; diet: dietary recalls	Participants received written educational materials that included recommendations for daily exercise and the Dietary Guidelines for Americans 2005 as well as telephone counseling from a registered dietitian trained in motivational interviewing techniques.	Received written educational materials	Social cognitive theory
Gallagher et al [43], 2022; Australia	Patients with coronary heart disease	n=390; age: mean 61.2 (SD 11.5) y; 17.5% women	PA; smoking	2 arms; 6 mo; self-reported	Mobile app	PA: GPAQ	The intervention was a gamification-based mobile app that encouraged users to engage in a healthy lifestyle and risk factor change through elements such as tracking behaviors, setting short- and long-term challenges, games, and quizzes; the game elements incorporated social cognitive theory strategies to promote sustained behavior change through message prompts, game challenges, and rewards.	Usual care	Social cognitive theory
Glasgow et al [44], 2012 United States	Patients with type 2 diabetes mellitus with obesity	n=204; age: mean 58.4 (SD 9.2) y; 49.8% women	PA; SB^g^	3 arms; 12 mo; NR	Website-based	PA: CHAMPS^h^ questionnaire; SB: NR	Participants were given access to the health website and asked to select goals in 3 areas (medication adherence, PA, and food choices), and they received periodic motivational calls; they recorded their progress and received immediate feedback on their success in meeting their goals over the past 7 days.	Usual care	Behavior change theory; social cognitive theory; social ecological theory
Golshahi et al [45], 2015; Iran	Patients with hypertension	n=822; age: mean 57.35 (SD 8.32) y; 78% women	PA; smoking; diet	4 arms; 5 mo; 8 times in total	SMS text messages	Self-report	Patients were advised to adhere to taking medication daily; increase PA (most days of the week); follow the dietary approach to prevent and manage hypertension (DASH^i^ diet), including eating a diet rich in vegetables and reducing dietary sodium to <1500 mg/d; and stop smoking.	Usual care	NR
Grey et al [46], 2019; United Kingdom	Adults with overweight or obesity	n=60; age: mean 50 (SD 8.9) y; 44.1% women	PA; SB; diet	2 arms; 3 mo; NR	Website-based	PA and SB: BodyMedia SenseWear core monitors; diet: 3-day weighed food and fluid records	The intervention was web-based and provided participants with information about PA and healthy eating, as well as a personalized interactive area for participants to set goals and plans related to PA and eating and to monitor their progress toward achieving their goals.	Face-to-face introductory session and NHS^j^ online health resources	Behavior change techniques
Harrigan et al [47], 2016; United States	Survivors of breast cancer with a BMI of 25.0 kg/m^2^	n=100; age: mean 59.0 (SD 7.5) y; 100% women	PA; diet	3 arms; 6 mo; NR	Telephone	PA: interview-administered PA questionnaire and pedometers; diet: FFQ	This intervention was composed of 11 coaching sessions (each lasting 30 min), represented by a social core curriculum with specific information about nutrition, exercise, and behavior strategies.	Usual care	Social cognitive theory
Hawkes et al [48], 2013; Canada	Patients with colorectal cancer	n=410; age: mean 66.4 (SD 10.6) y; 53.9% women	PA; alcohol use; diet	2 arms, 6 mo; 11 times in total	Telephone	PA: Godin Leisure Time Exercise Questionnaire; diet: FFQ	The intervention was conducted with 11 telephone health coaching sessions over 6 months, and participants were given manuals, motivational postcard prompts, and pedometers; the telephone sessions addressed lifestyle health behaviors and strategies, while the participant handbook included educational information on healthy behaviors.	Usual care	Behavior change techniques
Jane et al [49], 2017; Australia	Adults with overweight and obesity	n=67; age: mean 50.0 (SD 11.2) y; 85% women	PA; alcohol use; diet	3 arms; 3 mo; NR	Social networking tool	Diet: 3-day food records	Participants received a condensed version of the diet, which included detailed information and instructions.	Usual care	NR
Jennings et al [50], 2014; Australia	Patients with type 2 diabetes mellitus	n=397; age: mean 58.2 (SD 10.3) y; 47.6% women	PA; SB	2 arms; 3 mo; NR	Web -based	PA and SB: IPAQ, long-form version; pedometers	The website program, which used a self-management approach and was developed based on the theory of planned behavior, started a new health module topic each week, and weekly email reminders were distributed to participants; the content changed every week but always included a link to the intervention website.	Waitlist	Theory of planned behavior
Johnston et al [51], 2016; Sweden	Patients with myocardial infarction	n=174; age: mean 57.6 (SD 8.3) y; 18.4% women	PA; smoking	2 arms; 6 mo; every day	Mobile app	PA: PA questionnaires	Participants received a complete interactive patient support tool (web-based application) installed on their smartphones; educational information was sent to participants, including an extended drug adherence e-diary as well as exercise, weight, and smoking modules.	Usual care plus a simplified tool	NR
Kanera et al [52], 2017; Netherlands	Survivors of early cancer diagnoses	n=462; age: mean 55.9 (SD 11.4) y; 79.9% women	PA; diet	2 arms; 6 mo; NR	Web-based	PA: SQUASH^k^; diet: Dutch Standard Questionnaire on Food Consumption	The intervention was a web-based self-management program that collected self-reported lifestyle scores and compared them to guidelines, including advice on what modules (eg, PA and diet modules) were most relevant for them to use; 4 weeks after completing a module, participants were invited to participate in a brief online personalized assessment session; they were given personalized feedback to increase levels of coping self-efficacy to improve behavior maintenance.	Usual care	Theory of planned behavior; self-regulation theory
Krebs et al [53], 2017; United States	Survivors of cancer	n=86; age: mean 59.8 (SD 11.4) y; 82.96% women	PA; diet	2 arms; 3 mo; NR	DVD	PA: Godin Leisure Time Exercise Questionnaire; diet: FFQ	The intervention, which was based on formative evaluation data from a patient telephone survey, was delivered via a DVD; the DVD program included healthy eating and PA recommendations and took approximately 60 minutes to complete.	Usual care plus brief counseling	Social cognitive theory
Lara et al [54], 2016; United States	Retired adults	n=75; age: mean 61 (SD 4) y; 75% women	PA; diet	2 arms; 8 wk; mean 11.4 times in total	Web-based	PA: pedometer; accelerometer; diet: 24-hour recall method	The intervention comprised 5 modules (time, changing work, moving more, being social, and eating well) as well as a diary and a dashboard section to assist with site navigation; the intervention content was personalized to the participant based on information provided during use.	Usual care	Behavior change techniques
Li et al [55], 2022; United States	Patients with insomnia symptoms	n=21; age: mean 73.3 (SD 6.6) y; 71.4% women	PA; sleep	2 arms; 6 mo; 2 times/mo	Smartphone and smartwatch	PA: Actiwatch 2; PASE^l^; sleep: PSQI^m^	The intervention included (1) mHealth^n^ technology learning sessions, (2) personalized PA training, (3) mHealth strategies for PA, (4) financial incentives for completing the prescribed PA, and (5) additional support for mHealth technology.	Program book and a brief 1-time educational session	NR
Liu et al [56], 2018; Canada	Patients with hypertension	n= 129; age: mean 56.9 (SD 9.05) y; 47.7% women	PA; diet	3 arms; 4 mo; voluntary sign-in	Web-based	PA: pedometer; diet: Block Food Frequency Questionnaire	Participants set their own goals or chose interventions to achieve behavioral goals through e-counseling; the user-driven e-consultation group provided weekly information via email and options for areas of lifestyle change through text and video web links that contained information on developing exercise and diet plans, setting behavioral goals, self-monitoring lifestyle behaviors and blood pressure, resolving psychological conflicts, increasing change efficacy, and reviewing social and cognitive behavioral skills for relapse prevention to help participants maintain adherence.	A weekly email newsletter	Transtheoretical model
Morey et al [57], 2009; Canada, United Kingdom, and United States	Survivors of cancer	n=641; age: mean 73.1 (SD 5.1) y; 54.6% women	PA: NR; diet	2 arms; 12 mo; 15 sessions and 8 prompts	Telephone	PA: CHAMPS questionnaire; diet: 24-hour recalls	Participants received a personalized workbook containing advice on lifestyle and weight status; they also received aids such as pedometers, exercise bands, exercise posters, and a food portion schedule guide; each participant was assigned a health counselor to provide support and advice during counseling; in addition, the principal investigator provided support services over the telephone and sent regular progress reports to participants to motivate them to continue to change their behavior.	None	Social cognitive theory; transtheoretical model
Müller et al [58], 2016; Australia	Older Malaysians	n=43; age: mean 63.28 (SD 4.50) y; 74% women	PA; SB	2 arms; 3 mo; 5 times/wk	Text messages	PA and SB: IPAQ, short-form version	Pamphlets were distributed to participants. Participants were introduced specific exercises and provided a printed home-based exercise booklet additional text messages were sent to encourage and remind participants to follow the exercise program.	Exercise booklet	Behavior change techniques
Pischke et al [59], 2022; Germany	Adults aged ≥60 y	n=204; age: mean 68.7 (SD 5.4) y; 66.2% women	PA; SB	3 arms; 6 mo; NR	Web-based	PA and SB: accelerometer	Intervention materials were provided via a website. participants were asked to provide weekly feedback on PA goal completion; weekly group sessions provided to participants included performing the exercises in groups, going for joint walks, and discussing weekly health education topics.	A print intervention with subjective PA self-monitoring via printed PA pyramid	Self-regulation theory and various behavior change techniques
Poppe et al [60], 2019a; Belgium	Patients with type 2 diabetes mellitus	n=54; age: mean 62.67 (SD 8.4) y; 37% women	PA; SB	3 arms; 5 wk; 1 time/wk	Mobile app	PA and SB: IPAQ; accelerometer	The intervention developed a personal action plan to change selected health behaviors, anticipated potential barriers and found solutions, and enabled users to choose how to monitor their behavior; at the end of the first session, the user’s responses were summarized in a printable action plan, and they were provided with optional information on how they could obtain support from a partner, friend, family member, or colleague.	Waitlist	Behavior change theory
Poppe et al [60], 2019b; Belgium	Adults aged ≥50 y	n=63; age: mean 58.68 (SD 7.76) y; 75% women	PA; SB	3 arms; 5 wk; 1 time/wk	Mobile app	PA and SB: IPAQ; accelerometer	The intervention developed a personal action plan to change selected health behaviors, anticipated potential barriers and found solutions, and enabled users to choose how to monitor their behavior; at the end of the first session, the user’s responses were summarized in a printable action plan, and they were provided with optional information on how they could obtain support from a partner, friend, family member, or colleague.	Waitlist	Behavior change theory
Prabhakaran et al [61], 2019; India	Patients with hypertension or diabetes mellitus	n=3698; age: mean 55.1 (SD 11.0) y; 44.8% women	smoking; alcohol use	2 arms; 12 mo; NR	Mobile app	Self-reporting	The intervention stored the health records electronically, enabling long-term monitoring and follow-up; it was also equipped to send SMS text message reminders (to take medication and attend follow-up visits) to patients.	Usual care	NR
Sakane et al [62], 2023; Japan	Patients with obesity and hypertension	n=78; age: mean 51.95 (SD 6.5) y; 0% women	PA; diet; smoking	2 arms; 3 mo	Mobile app	Self-reporting	The app provided an initial assessment of the participant, identified a behavioral agenda (exercise, diet, lifestyle habits, and number of steps per day), and enabled self-monitoring.	Usual care	Behavior change techniques
Swoboda et al [63], 2016; United States	Patients with type 2 diabetes mellitus	n=60; age: mean 56.1 (SD 6.7) y; 68.5% women	PA; SB; diet	3 arms; 4 mo; biweekly telephone calls (7 calls in total)	Telephone	PA and SB: IPAQ, long-form version; diet: FFQ	Self-set goals related to diet and PA were developed based on teleconferences, and action plans were developed to help participants achieve their goals; motivational interviewing and decision-making guidance were used to help participants develop individualized diet and activity goals.	Attention control	NR
Taylor et al [64], 2021; United Kingdom	Patients with inactive exercisers and chronic health conditions	n=450; age: mean 50 (SD, 12) y; 64% women	PA; SB; sleep	2 arms; 4 mo; NR	Web-based	PA and SB: accelerometer and pedometer; sleep: accelerometer	The intervention (e-coachER) offered a range of interactive opportunities to enhance participants’ motivation to take up the exercise referral scheme and to maintain a more physically active lifestyle, whether or not they engaged with their local exercise referral scheme; participants were encouraged to make use of the pedometer and the activity record sheets for self-monitoring and goal setting in conjunction with the e-coachER website.	Usual care plus an exercise referral scheme	Self-determination theory
Wang et al [65], 2020; China	Patients with type 2 diabetes mellitus	n=171; age: mean 55.1 (SD 10.8) y; 43% women	PA; smoking; alcohol use	2 arms; 12 mo; 2 times/wk	SMS text messages	Self-reporting	The information design included 5 main areas: health awareness, dietary control, PA, lifestyle habits, and weight control; telephone follow-up was conducted after each stage of the intervention.	Regular education plus general theoretical knowledge messages	Transtheoretical model
Watson et al [66], 2015; United Kingdom	Adults who were obese and inactive with ≥1 CVD^o^ risk factors	n=65; age: mean 52.1 (SD 7.4) y; 55% women	PA; diet	2 arms; 12 mo; personalized information service	Web-based	PA: validated RPAQ^p^; diet: 3-month recall	The Imperative Health system generated personalized daily targets (weight loss, PA, and dietary targets) for each participant; automated weekly feedback on the participants’ performance, assessed by the self-monitoring devices (weighing scales and accelerometer) and the food diary, was provided.	Usual care	Social support and decisional balance theory
Zheng et al [67], 2019; China	Patients with coronary heart disease and without diabetes mellitus	n=822; age: mean 56.4 (SD 9.5) y; 14.1% women	PA; smoking	2 arms; 6 mo; 6 times/wk	Text messages	PA: IPAQ; short-form version	The intervention was designed as a secondary prevention program that sent participants 1 of each of the following text message types according to a prespecified algorithm: general knowledge of coronary heart disease, blood pressure control, medication adherence, PA, healthy eating, and smoking cessation.	Usual care plus 2 thank-you messages per month	Behavior change theory

^a^PA: physical activity.

^b^NR: not reported.

^c^IPAQ: International Physical Activity Questionnaire.

^d^TEXT ME: Tobacco, Exercise, and Diet Messages.

^e^FFQ: Food Frequency Questionnaire.

^f^GPAQ: Global Physical Activity Questionnaire.

^g^SB: sedentary behavior.

^h^CHAMPS: Community Healthy Activities Model Program for Seniors.

^i^DASH: Dietary Approaches to Stop Hypertension.

^j^NHS: National Health Service.

^k^SQUASH: Short Questionnaire to Assess Health-Enhancing Physical Activity.

^l^PASE: Physical Activity Scale for the Elderly.

^m^PSQI: Pittsburgh Sleep Quality Index.

^n^mHealth: mobile health.

^o^CVD: cardiovascular disease.

^p^RPAQ: Recent Physical Activity Questionnaire.

#### Characteristics of Participants

A total of 12,931 participants were enrolled in the included studies, with the number of participants ranging from 21 to 879 in individual studies. The mean age of the older adults included ranged between 50.0 (SD 11.2) years [49] and 73.3 (SD 6.6) years [55]. Nearly one-half of the participants (6009/12,931, 46.47%) were female, and 2 (6%) of the 35 studies targeted only women [42,47]. The studies included not only healthy older adults (4/35, 11%) but also people with various chronic conditions, such as cancer (8/35, 23%), type 2 diabetes mellitus (6/35, 17%), hypertension (4/35, 11%), coronary disease (4/35, 11%), and overweight or obesity (3/35, 9%).

#### Characteristics of eHealth Interventions

The eHealth interventions included in these studies were delivered mainly through 8 intervention methods: SMS text message [36,40,45,58,65,67], telephone [35,39,42,47,48,55,57,63], email [41], website [37,50,52,54,56,59,64,66], mobile app [44,51,60-62], social tool [38,49], DVD [53], and smartwatch [55]; and the most common methods were based on a website (8/35, 23%), SMS text message (6/35, 17%), and mobile app (6/35, 17%). Intervention durations in the included studies ranged from 5 weeks to 21 months, the most common being 6 months (12/35, 34%) and 12 months (8/35, 23%).

Most of the studies (30/35, 86%) targeted 2 to 3 risk factors. Of the 35 studies, 5 (14%) investigated 4 (67%) of the 6 behaviors (PA, smoking cessation, alcohol cessation, and diet) [35,38,41], and only 9% (3/35) of the studies addressed sleep [37,55,64]. The most common combination was PA and diet (8/35, 23%), followed by PA and SB (7/35, 20%) and PA and smoking cessation (5/35, 14%). Of the 35 studies, 25 (71%) used a theoretical basis (n=18, 72%) or behavior change techniques (n=7, 28%). In the studies using theoretical underpinnings, the most common were the social cognitive model (7/25, 28%) and behavior change theory or techniques (12/25, 48%); the studies also used the transtheoretical model, the theory of planned behavior, the self-regulation model, and the self-determination model.

#### Characteristics of Controls

All participants in the control groups received usual care during the study period. In addition, 6 (17%) of the 35 studies supplied a paper version of the guidance booklet [39,42,55,58,59,64]. Zheng et al [67] and Wang et al [65] sent thank-you text messages or general theoretical knowledge information, Grey et al [46] provided face-to-face introductory sessions and National Health Service online health resources, and Swoboda et al [63] conducted an attention control intervention.

#### Outcome Measures

A variety of outcomes were subjectively or objectively assessed before and after the intervention. The measurement tools used for the different outcomes are shown in Multimedia Appendix 3.

The end points of self-reported total PA (TPA), objectively measured TPA, self-reported moderate to vigorous PA (MVPA), objectively measured MVPA, daily steps, regular exercise, and inactive PA were used to describe PA behavior. More than 10 different questionnaires were used to assess participants’ self-reported PA levels and SB levels, the most common of which was the International Physical Activity Questionnaire. Objective measurements of TPA and MVPA were obtained from accelerometer measurements, and a pedometer was used to measure daily steps. Regular exercise was defined as accumulating >30 minutes of moderate exercise performed ≥5 days per week. PA was categorized into active and inactive on the basis of established recommendations regarding the total level: ≥600 and <600 metabolic equivalent of task minutes per week, respectively. Eight indicators were selected to describe diet behavior: energy intake as well as intake of fruits, vegetables, fruits and vegetables (together), fats, proteins, sugars, and fiber. Of the 35 studies, 17 (49%) used various versions of the Food Frequency Questionnaire and dietary records or recall from different periods to evaluate the dietary indicators. In addition, the Dutch Standard Questionnaire on Food Consumption was used in the study by Kanera et al [52]. The 3 studies involving sleep used 3 measuring tools, namely the 5-item validated Women’s Health Initiative Insomnia Rating Scale, the Pittsburgh Sleep Quality Index, and an accelerometer. The prevalence of alcohol and tobacco use was collected through interviews or questionnaires.

#### Risk of Bias

The included studies were assessed for risk of bias using the risk-of-bias tool for randomized trials in the Cochrane Handbook for Systematic Review of Interventions (version 5.1.0). A summary of the overall risk-of-bias assessment for all included studies as well as the judgments regarding specific studies can be found in Multimedia Appendix 4 [34-67].

According to our assessment, which was conducted by 2 independent reviewers (BS and GL), 17 (49%) of the 35 publications had a high risk of bias for not blinding participants and study personnel to the intervention assignment; however, blinding in intervention trials for behavior change is challenging. For most of the publications (20/35, 57%), the risk associated with allocation concealment was high (6/35, 17%) or unclear (14/35, 40%). Of the 35 studies, 9 (26%) did not provide sufficient detail to determine whether researchers were blinded to the assessment of outcomes; therefore, the risk was rated as unclear.

#### Certainty of Evidence

Of all outcomes listed in the summary of findings presented in Multimedia Appendix 5, we deemed the quality of the evidence to be very low to high, as determined by the GRADE framework. We judged the quality of evidence for most of the diet-related outcome indicators to be high. The evidence for outcome indicators related to PA and SB was low, mainly because of the high risk of bias, inconsistent study results, and imprecise measurements. In addition, evidence relating to smoking, alcohol use, and sleep was deemed to be very low or low because of the high risk of bias, small sample sizes, and imprecise measurements.

### Meta-Analysis Results

#### Overview

**Figure 2:**
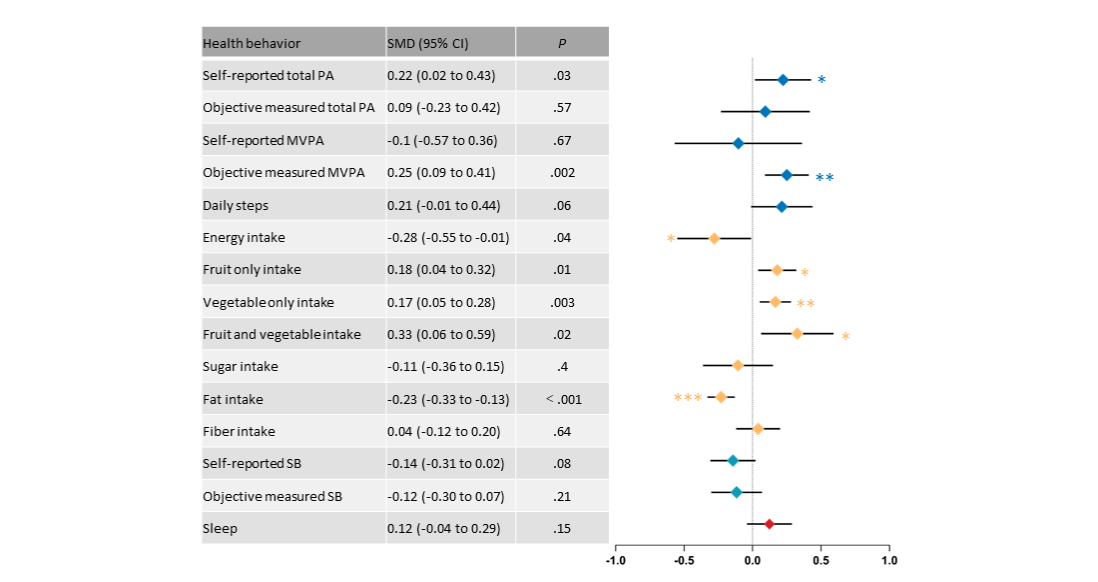
Forest plot integration for continuous outcomes. MVPA: moderate to vigorous physical activity; PA: physical activity; SB: sedentary behavior; SMD: standard mean difference. *P<.05, **P<.01, ***P<.001.

**Figure 3:**
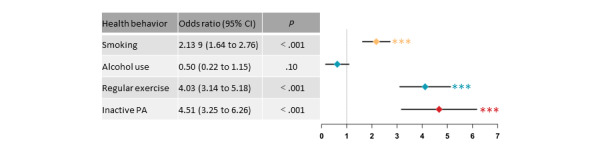
Forest plot integration for dichotomous outcomes. PA: physical activity. ***P<.001.

We have summarized the forest plot results for continuous (Figure 2) and dichotomous (Figure 3) variable outcomes. Overall, eHealth-based MHBC interventions may be able to improve PA, diet, and smoking cessation in older adults, with no effect on alcohol use, SB, or sleep. Forest plots for each of the outcomes are presented in Multimedia Appendix 6 [34-67].

#### PA Assessment

The effectiveness of eHealth interventions on self-reported TPA levels in older adults was assessed in 17 (49%) of the 35 studies. Compared with the control group, the eHealth interventions significantly increased self-reported TPA (SMD 0.22, 95% CI 0.02-0.43; *P*=.03), with substantial heterogeneity between the studies (*I*^2^=79%; *P*<.001; Figure S1A in Multimedia Appendix 6). The sensitivity analysis suggested a similar result (SMD 0.12, 95% CI 0.04-0.20; *P*=.006), and the heterogeneity dropped significantly (*I*^2^=0%; *P*=.80) after excluding the study by Cicolini et al [41] (Multimedia Appendix 7 [34-67]). A meta-analysis of the effectiveness of eHealth interventions on objectively measured TPA by a fixed effects model (*I*²=19%; *P*=.79) showed the opposite result (SMD 0.09, 95% CI –0.23 to 0.42; *P*=.57; Figure S1B in Multimedia Appendix 6). No significant increase was observed in self-reported MVPA after the eHealth intervention (SMD 0.10, 95% CI –0.57 to 0.36; *P*=.67), with greater heterogeneity (*I*²=94%; *P*<.001; Figure S1C in Multimedia Appendix 6). After excluding the studies by Taylor et al [64] and Pischke et al [59], synthesis using fixed effects models showed a significant increase in self-reported MVPA after the intervention (SMD 0.15, 95% CI 0.03-0.27; *P*=.02), with moderate heterogeneity (*I*²=54%; *P*=.03). However, the eHealth-based MHBC interventions led to a small but significant increase in objectively measured MVPA (SMD 0.25, 95% CI 0.09-0.41; *P*=.002), without any heterogeneity (*I*²=0%; *P*=.76; Figure S1D in Multimedia Appendix 6). In addition, a meta-analysis of the 7 studies reporting daily steps showed that the eHealth interventions increased the number of steps taken by older adults compared to the control group (SMD 0.21, 95% CI –0.01 to 0.44; *P*=.06), corresponding to an average estimate of 597 steps, with borderline significant results and low heterogeneity (*I*²=0%; *P*=.64; Figure S1E in Multimedia Appendix 6). Overall, compared to the controls, the eHealth interventions significantly increased the prevalence of regular exercise (OR 4.17, 95% CI 3.42-5.36; *P*<.001), with low heterogeneity (*I*²=0%; *P*<.001; Figure S1F in Multimedia Appendix 6); and significantly reduced the prevalence of inactive PA (OR 4.53, 95% CI 3.27-6.28; *P*<.001), with low heterogeneity (*I*²=16%; *P*=.31; Figure S1G in Multimedia Appendix 6). Sensitivity analysis showed that these results were relatively stable.

#### SB Assessment

Of the 35 studies, 9 (26%) assessed self-reported SB and 7 (20%) assessed objectively measured SB. A meta-analysis showed no effect of the eHealth intervention on self-reported sedentary time compared to the controls (SMD –0.14, 95% CI –0.31 to 0.02; *P*=.08; *I*²=0%; *P*=.95; Figure S2A in Multimedia Appendix 6). Similarly, there was no effect of the eHealth intervention on objectively measured sedentary time (SMD –0.12, 95% CI –0.30 to 0.07; *P*=.21; *I*²=0%; *P*=.91; Figure S2B in Multimedia Appendix 6). Sensitivity analysis showed that the result was stable.

#### Diet

Mean energy intake (kcal) data from 9 (26%) of the 35 trials were suitable for meta-analysis. Energy intake was significantly lower in the eHealth intervention group (SMD –0.28, 95% CI –0.55 to –0.01; *P*=.04) compared to the control group, with low heterogeneity (*I*²=0%; *P*=.54; Figure S3A in Multimedia Appendix 6). Of the 35 trials, 8 (23%), 10 (29%), and 7 (20%) assessed intake of fruits (servings), vegetables (servings, portion, and times), and fruits and vegetables (servings), respectively, and the eHealth interventions significantly increased intake of fruits (SMD 0.18, 95% CI 0.04-0.32; *P*=.01; *I*²=19%; *P*=.28; Figure S3B in Multimedia Appendix 6), intake of vegetables (SMD 0.17, 95% CI 0.05-0.28; *P*=.003; *I*²=12%; *P*=.34; Figure S3C in Multimedia Appendix 6), and intake of fruits and vegetables (SMD 0.18, 95% CI 0.04-0.32; *P*=.01; *I*²=19%; *P*=.28; Figure S3D in Multimedia Appendix 6) compared to the controls. Of the 35 trials, 5 (14%) assessed sugar intake, which did not change significantly after the intervention compared to the control group (SMD –0.11, 95% CI –0.36 to 0.15; *P*=.40; *I*²=0%; *P*=.45; Figure S3E in Multimedia Appendix 6). Of the 35 trials, 9 (26%) assessed fat intake changes, and there was a significant reduction in fat intake after the eHealth intervention (SMD –0.23, 95% CI –0.33 to –0.13; *P*<.001; *I*²=0%; *P*=.93; Figure S3F in Multimedia Appendix 6). Similarly, a meta-analysis of the 7 trials that assessed fiber intake showed no significant changes (SMD 0.04, 95% CI –0.12 to 0.20; *P*=.64; *I*²=14%; *P*=.32; Figure S3G in Multimedia Appendix 6). Sensitivity analysis showed that the result was relatively stable.

#### Smoking and Alcohol Use

A meta-analysis of the 8 studies reporting outcomes on smoking showed that the eHealth interventions significantly reduced the prevalence of smoking (OR 2.09, 95% CI 1.62-2.70; *P*<.001) but with greater heterogeneity (*I*^2^=85%; *P*<.001; Figure S4A in Multimedia Appendix 6). After excluding the studies by Gallagher et al [43] and Prabhakaran et al [61], sensitivity analysis showed that the result was stable (OR 3.64, 95% CI 2.68-4.93; *P*<.001), and the results were homogeneous across the trials (*I*^2^=0%; *P*=.76). Of the 35 studies, 4 (11%) reported outcomes for alcohol use, and meta-analysis showed no effect of the eHealth intervention on the prevalence of alcohol use (OR 0.75, 95% CI 0.26-2.18; *P*=.60), with less heterogeneity (*I*²=65%; *P*=.03; Figure S4B in Multimedia Appendix 6). After excluding the study by Babu et al [35], sensitivity analysis showed that the result was stable (OR 0.50, 95% CI 0.22-1.15; *P*=.10), and the results were homogeneous across the trials (*I*^2^=40%; *P*=.19).

#### Sleep

Data from 3 (9%) of the 35 studies were used to assess the effectiveness of eHealth interventions on sleep in older adults. Due to the low level of article heterogeneity (*I*^2^=44%; *P*=.17) and the small number of included studies, a fixed effects model was used. Meta-analysis showed that the eHealth interventions did not affect sleeping time in older adults (SMD 0.12, 95% CI –0.04 to 0.29; *P*=.15; Figure S5 in Multimedia Appendix 6). Sensitivity analysis showed that the result was stable.

## Discussion

### Overview

This systematic review is the first study to systematically examine the effectiveness of eHealth interventions targeting ≥2 of the following behaviors in a population of older adults (aged ≥50 y): PA, diet, smoking, alcohol consumption, sleep, and SB. A total of 35 studies with 12,931 older adults aged ≥50 years met the eligibility criteria for this review. The majority (6009/12,931, 46.47%) of the participants were female, with a mean age ranging from 50.0 (SD 11.2) years to 73.3 (SD 6.6) years. The overall methodological quality was moderate, according to the GRADE framework. Compared to usual care or waitlist, eHealth-based MHBC interventions significantly changed diet, SB, and smoking but had little effect on PA, alcohol consumption, and sleep.

### Principal Findings

#### PA Assessment

Our data suggest that eHealth-based MHBC interventions may improve PA levels and daily steps in older adults. Previous meta-analyses also revealed that eHealth-based MHBC interventions significantly promoted PA among people with noncommunicable diseases and adults compared to the control conditions [22,68]. It is worth noting that the PA referred to in both studies was a synthetic size of multiple effect sizes within a particular scope of outcomes (eg, energy expenditure, steps, and time spent in PA or MVPA). We conducted a separate meta-analysis of PA-related metrics. First, the eHealth-based MHBC intervention significantly improved older adults’ self-reported TPA compared with the control group. This is consistent with a previous meta-analysis [28], which found a mean increase in TPA of 90.7 minutes per week according to questionnaires in the eHealth-based MHBC intervention group compared to the control group. Another review involving people aged ≥50 years found that interventions containing smart technology significantly increased self-reported TPA compared to face-to-face interventions (SMD 0.17, 95% CI 0.02-0.32) [69], which is broadly comparable to our effect size. Second, although we also found that the eHealth-based MHBC intervention significantly increased the time spent in MVPA in older adults, this result needs to be interpreted with caution because 4 (80%) of the 5 data points are from the same article. Interestingly, this review found very different results for self-reported and objectively measured TPA or MVPA. Another review reported similar results, with eHealth interventions significantly increasing objectively measured MVPA but not self-reported MVPA [69]. This may be because the MHBC intervention intervened on a larger number of target behaviors than the SHBC intervention, and the intervention effect was unstable [70,71]. Another reason may be that the effect of eHealth interventions on the PA time measured by ActiGraph devices and that measured by questionnaires was quite different [72]. Third, this review also showed that participants in the eHealth intervention group walked more than those in the control group (mean difference 597 steps/d). This is a conclusion we believe to be quite credible [28,69]. Earlier studies have shown that older people commonly cite inconvenience and a lack of access to a PA program as reasons for avoiding performing PA [73]. Walking is the most commonly targeted PA for older people because it does not require access to specific programs and can be practiced anywhere [74]. Finally, we also meta-analyzed changes in the prevalence of regular exercise as well as inactive PA and found a positive effect of an eHealth-based MHBC intervention. With up to 40% of people in industrialized countries not engaging in any regular PA [75], increasing PA rates by the rate produced by these interventions would have an important impact on health outcomes.

#### Diet

Our review found that all MHBC interventions involving dietary interventions in older adults were performed together with PA, with a combination of both of them being the most common (8/35, 23%). This combination was also found by previous authors to be the most common combination in adolescent and adult MHBC interventions [26,68]. The results of our study suggest that eHealth-based MHBC interventions can significantly improve the diet of older adults. Specifically, there was an increase in fruit or vegetable intake. This finding aligns with previous research demonstrating that both the eHealth-based SHBC intervention and the eHealth-based MHBC intervention significantly boosted fruit or vegetable intake in the intervention group [26,53,76]. Furthermore, we also found significant reductions in fat intake and energy intake in this population. eHealth interventions similarly significantly reduced dietary fat intake in adults but not in adolescents [26,76]. This may be because the health benefits of reducing fat intake are greater for middle-aged and older adults; therefore, they have greater motivational self-efficacy [77]. eHealth interventions have an impact not only on dietary intake (33%-35%) but also on diet-related cognitive variables (69%-79%) [78]. In addition, we found no evidence that eHealth interventions can change the dietary intake of sugar and fiber in older people. Although other studies have shown that it is possible to reduce children’s consumption of sugary beverages, reducing unhealthy eating behaviors is often considered more difficult than starting new healthy behaviors [79,80]. The contents of eHealth interventions that are communicated to participants about behavior change and the feedback they receive can also influence changes in participants’ behavior. Further research is needed to examine intervention contents regarding these dietary outcomes to increase the size and sustainability of the effects of MHBC interventions, particularly among older adults.

#### Smoking and Alcohol Use

The effectiveness of eHealth-based MHBC interventions for stopping drinking is generally weak; however, promising results were seen for smoking cessation. The quality of evidence for alcohol use was deemed to be very low because the review only included 4 RCTs with relatively small sample sizes, which might have affected the pooled effect. In general, the longer the duration of the drinking and smoking interventions, the more effective they are [81]. In this review, we included studies with intervention durations of 3 months as well as 6 months, in addition to the 12-month intervention duration. Due to the limited number of studies, we did not conduct further analyses. Indeed, the evidence for the effectiveness of eHealth-based smoking and alcohol cessation interventions is currently controversial [82,83]. There is also evidence that eHealth interventions may have similar effects in reducing drinking and smoking when compared to face‐to‐face treatment or no support [84,85]. Moreover, SMS text messaging may be particularly powerful for smoking behaviors such as abstinence and reduction of use, particularly automated reminders and motivating messages that can be sent during times of cravings [86]. Thus, further work is needed to detect specific moderators of interventions such as follow-up length and message frequency.

#### SB Assessment

The lack of a significant reduction in either self-reported sedentary time (*P*=.08) or objectively measured sedentary time (*P*=.06) was an unexpected finding, leaving ample room for improvement. Champion et al [26] found that eHealth-based MHBC interventions led to decreased sedentary time compared to controls (SMD –0.09, 95% CI –0.17 to –0.01). Although in the same direction and a similar effect size to our result, their result was statistically significant. Previous literature suggests that eHealth interventions can improve sedentary time within, but not between, groups of older adults. The review by Yerrakalva et al [87] showed that mobile health app interventions had the potential to reduce SB and increase PA in older adults. The MHBC studies for SB included in this review are all combined with PA, and most of the comments received by participants in the eHealth interventions (via SMS text messaging and telephone) supported their focus on PA and the step goals rather than on SB. In addition, research suggests that given the automaticity of SB, different and more effortful strategies are required to break existing habits compared to forming new habits [88,89]. This hints that future interventions regarding MHBC interventions could use multiple intervention strategies to reduce sedentary time [90].

#### Sleep

Sleep duration is a commonly used indicator for assessing sleep quality, and suboptimal sleep duration has been associated with increased morbidity and mortality [91], with short sleep durations in particular being detrimental to health [92]. We found that eHealth-based MHBC interventions may not significantly improve sleep duration in older adults. Of the 3 studies on sleep that we included, 2 (67%) found significant improvements in sleep quality. However, the third study did not find that the eHealth intervention did not improve sleep duration. This may be because, in this study, sleep was used as a secondary outcome, and the intervention only set specific PA goals for the participants without any recommendations for sleep. In contrast to our findings, Howarth et al [93] evaluated the impact of a workplace digital health intervention on health-related outcomes and found that it could improve participants’ sleep quality. Inconsistencies in conclusions may be due to possible differences in the effects of eHealth interventions on various indicators of sleep. In addition to objectively measured sleep duration, a range of subjectively measured sleep outcomes (nocturnal sleep quality, sleep duration, sleep efficiency, sleep latency, and sleep medication use) are equally important in older adults with insomnia or insomnia symptoms [94]. PA resulted in significant improvements in overall sleep quality, sleep quality, and sleep latency but not in sleep duration, efficiency, or disturbance [95]. According to the American National Sleep Foundation, nonpharmacological treatment options are the preferred first choice of treatment for sleep problems [96]. Therefore, the doubt whether MHBC interventions can improve sleep problems in older adults is important and needs to be explored in more RCTs.

### Strengths and Limitations

The strengths of this study include the focus on a variety of lifestyle behaviors associated with adverse health outcomes and our rigorous methodology.

However, our study has several limitations. First, despite our best efforts to conduct a thorough literature search in a limited number of databases, the diversity of outcome indicator descriptions may still have resulted in the omission of appropriate topics or relevant studies. Second, this review included some participants aged 50 to 60 years because some of the trials were designed to recruit older adults, but they did not specifically exclude people aged <60 years. Future research should explicitly target older adults (aged ≥60 y) for RCTs of eHealth interventions. Third, although all included studies used eHealth interventions, there was still considerable variation in terms of participants (eg, cultural background, age, and health status), intervention characteristics (eg, intervention channel, content, and duration), and outcome measures, and the small number of studies may lead to cautious interpretations of the pooled results. In particular, SHBC interventions differ from MHBC interventions in terms of effectiveness. Finally, because we only searched for articles published in English, the results of the meta-analysis may be affected by language bias.

### Implications

This paper highlights important directions for future research. First, there is a lack of qualified interventions to address sleep problems, although many older adults report sleep deprivation or sleep problems [97], and there is a growing recognition that exercise behaviors (such as PA), SB, and sleep are interdependent. Second, there is a general lack of follow-up analyses in intervention designs. Most of the included studies measured outcomes twice (before the intervention and after the intervention). Thus, the long-term and maintenance effects of eHealth interventions on multiple risk behavior changes in older adults have not been validated. As advocated [6,13], the next phase of this study is to explore how and under what conditions these initial changes can be maintained by adding a longer follow-up design. Finally, the intervention components included in the study focused primarily on health behavior education and counseling, with a lack of substantive behavioral coaching; this may be due to the limitations of current eHealth intervention channels. A commonly used intervention paradigm is to select a specific health behavior change theory (eg, behavior change theory, social cognitive theory, or self-regulation theory) as a framework and further promote a theory of choice for effective elements (eg, motivation, planning, and self-regulation). As the results of previous reviews have confirmed the effectiveness of this intervention paradigm, to further improve intervention effectiveness, it may be prudent to use dual-process approaches (ie, focusing on conscious and unconscious processes of behavior change) [54,56] alongside socioecological approaches (ie, involving policy dimensions, environment, and individual factors) [59,64].

In addition, we need to pay extra attention to the synergistic or transfer effects involved in MHBC interventions. Accordingly, lessons, skills, and knowledge learned concerning 1 behavior apply to another, thereby improving multiple behaviors. In addition, success in changing ≥1 lifestyle behaviors may also increase confidence or self-efficacy to improve risky behaviors in individuals who are less motivated to change [22]. In addition to potentially greater efficacy and impact on health, MHBC interventions have greater real-world applicability and provide information about the effective treatment of co-occurring behaviors [23]. This is particularly true among older adults, who are more likely to have co-occurring health conditions than younger adults. Future research should seek to explore this effect in trials of MHBC interventions.

### Conclusions

A wide range of eHealth-based MHBC interventions were effective in improving diet, aiding smoking cessation, and increasing daily step counts. However, effect sizes were small, and the overall quality of the evidence was low. Further high-quality research is needed to develop eHealth interventions that are effective in simultaneously modifying multiple risk factors for chronic disease, including substance use and sleep outcomes. It is recommended that eHealth-based MHBC interventions be included in the guidelines to improve the quality of life of older persons and reduce the risk of chronic diseases in later life.

## Appendix

Multimedia Appendix 1 PRISMA (Preferred Reporting Items for Systematic Reviews and Meta-Analyses) checklist.

Multimedia Appendix 2 Search strategy.

Multimedia Appendix 3 The measurement tools used for different outcomes.

Multimedia Appendix 4 Risk-of-bias summary.

Multimedia Appendix 5 GRADE (Grading of Recommendations Assessment, Development, and Evaluation) assessment results.

Multimedia Appendix 6 Forest plots.

Multimedia Appendix 7 Sensitivity analysis.

## Abbreviations

GRADE: Grading of Recommendations Assessment, Development, and Evaluation
MHBC: multiple health behavior change
MVPA: moderate to vigorous physical activity
OR: odds ratio
PA: physical activity
PICOS: Population, Intervention, Comparison, Outcomes, and Study Design
PRISMA: Preferred Reporting Items for Systematic Reviews and Meta-Analyses
RCT: randomized controlled trial
SB: sedentary behavior
SHBC: single health behavior change
SMD: standardized mean difference
TPA: total physical activity

## Data Availability

The data sets generated and analyzed during this study are available from the corresponding author on reasonable request.
