# Supplementary material for: Assessing the Effectiveness of eHealth Interventions to Manage Multiple Lifestyle Risk Behaviors Among Older Adults: Systematic Review and Meta-Analysis
Source: J Med Internet Res. 2024 Jul 31;26:e58174. doi: 10.2196/58174 (PMC11325121; doi:10.2196/58174)
Supplement: Multimedia Appendix 2 [file jmir_v26i1e58174_app2.docx]

**Multimedia Appendix 2: Search Strategy**

**Title:** Effectiveness of eHealth interventions to manage multiple lifestyle risk behaviors among older adults: a systematic review and meta-analysis

**The search ran:** 1 May 2025

**Databases:** PubMed, Embase, Web of Science, Scopus, Cochrane Library, and SPORT discus

| **Database** | **Search Strategies** | **Results** |
| --- | --- | --- |
| PubMed | #1 Search: (Aged[MeSH Terms]) OR (elder*[Title/Abstract] OR old*[Title/Abstract] OR senior*[Title/Abstract] OR veteran*[Title/Abstract] OR geriatric*[Title/Abstract] OR retire*[Title/Abstract])  #2 Search: (Internet-Based Intervention[MeSH Terms]OR Telemedicine[MeSH Terms] OR Telerehabilitation[MeSH Terms]) OR (ehealth[Title/Abstract] OR e-health[Title/Abstract] OR m-health[Title/Abstract] OR mhealth[Title/Abstract] OR mobile health[Title/Abstract] OR mobile communication[Title/Abstract] OR remote consult[Title/Abstract] OR telecommunication* [Title/Abstract] OR telehealth[Title/Abstract] OR tele health[Title/Abstract] OR telemedicine[Title/Abstract] OR tele medicine[Title/Abstract] OR telecare[Title/Abstract] OR tele-care[Title/Abstract] OR telehomecare[Title/Abstract] OR telehealth[Title/Abstract] OR tele-medicine[Title/Abstract] OR tele-homecare[Title/Abstract] OR telerehabilitation[Title/Abstract] OR tele-rehabilitation[Title/Abstract] OR tele-health[Title/Abstract] OR tele-monitor[Title/Abstract] OR telemonitor[Title/Abstract] OR tele-management[Title/Abstract] OR telemanagement[Title/Abstract] OR eletronic health[Title/Abstract] OR digital health[Title/Abstract] OR online health[Title/Abstract] OR on-line health[Title/Abstract] OR computer [Title/Abstract] OR internet based[Title/Abstract] OR internet [Title/Abstract] OR internet-based[Title/Abstract] OR web [Title/Abstract] OR web based[Title/Abstract] OR web-based[Title/Abstract] OR webcast*[Title/Abstract] OR mobile technology [Title/Abstract] OR mobile device [Title/Abstract] OR health app*[Title/Abstract] OR app* [Title/Abstract] OR application* [Title/Abstract] OR health applications [Title/Abstract] OR phone [Title/Abstract] OR cellphone [Title/Abstract] OR mobile phone [Title/Abstract] OR mobilephone [Title/Abstract] OR smartphone [Title/Abstract] OR smart phone [Title/Abstract] OR tablet [Title/Abstract] OR blog [Title/Abstract] OR blogging [Title/Abstract] OR SMS [Title/Abstract]OR short message servic* (health) OR text message[Title/Abstract] OR texting e-mail [Title/Abstract] OR email [Title/Abstract] OR computer [Title/Abstract] OR tablet [Title/Abstract] OR video conferenc* [Title/Abstract] OR video meeting [Title/Abstract] OR interactive video [Title/Abstract] OR TV meeting [Title/Abstract] OR serious gam*[Title/Abstract] OR video game* [Title/Abstract] OR videogame [Title/Abstract] OR gamification [Title/Abstract] OR digital game [Title/Abstract] OR gaming [Title/Abstract] OR social media [Title/Abstract] OR socialmedia [Title/Abstract] OR social network [Title/Abstract] OR virtual reality [Title/Abstract] OR VR [Title/Abstract] OR personal digital assistant[Title/Abstract])  #3 Search: (Health Behavior[MeSH Terms] OR Risk Reduction Behavior[MeSH Terms]) OR (health behavio*[Title/Abstract] OR health-related behavio*[Title/Abstract] OR health related behavio*[Title/Abstract] OR healthy lifestyle*[Title/Abstract] OR health promoting behavio*[Title/Abstract] OR risk reduction behavio*[Title/Abstract] OR risk behavio*[Title/Abstract] OR risky health[Title/Abstract])  #4 Search: (Randomized Controlled Trial[MeSH Terms]) OR (controlled clinical trial[Title/Abstract] OR randomized[Title/Abstract] OR randoml[Title/Abstract] OR trial[Title/Abstract])  # 5 Search: care-as-usual[Title/Abstract] OR control[Title/Abstract] OR no treatment[Title/Abstract] OR waitlist[Title/Abstract] OR wait-list[Title/Abstract] OR treatment-as-usual[Title/Abstract] OR paper[Title/Abstract] OR active control[Title/Abstract]  #6 Search: #1 AND #2 AND #3 AND #4 AND #5  #7 Search: Review[Title] OR Meta[Title] OR protocol[Title]  #8 Search: #6 NOT #7 | 1010 |
| Embase | #1 'aged'/exp  #2 'elder*':ab,ti OR 'old*':ab,ti OR 'senior*':ab,ti OR 'veteran*':ab,ti OR ' geriatric*':ab,ti OR 'retire*':ab,ti  #3 #1 OR #2  #4 'web-based intervention'/exp  #5 'telemedicine'/exp  #6 'telerehabilitation'/exp  #7 'telehealth'/exp  #8 'mhealth'/exp  #9 'telecare'/exp  #10 ‘internet-based intervention’:ab,ti OR ‘ehealth’:ab,ti OR ‘e-health’:ab,ti OR ‘mobile communication’:ab,ti OR ‘remote consult’:ab,ti OR ‘telecommunication*’:ab,ti OR ‘tele-homecare’:ab,ti OR ‘tele-monitor’:ab,ti OR ‘telemonitor’:ab,ti OR ‘tele-management’:ab,ti OR ‘telemanagement’:ab,ti OR ‘eletronic health’:ab,ti OR ‘digital health’:ab,ti OR ‘online health’:ab,ti OR ‘on-line health’:ab,ti OR ‘computer’:ab,ti OR ‘internet based’:ab,ti OR ‘internet’:ab,ti OR ‘internet-based’:ab,ti OR ‘webcast*’:ab,ti OR ‘mobile technology’:ab,ti OR ‘mobile device’:ab,ti OR ‘health app*’:ab,ti OR ‘app*’:ab,ti OR ‘application*’:ab,ti OR ‘health applications’:ab,ti OR ‘phone’:ab,ti OR ‘cellphone’:ab,ti OR ‘mobile phone’:ab,ti OR ‘mobilephone’:ab,ti OR ‘smartphone’:ab,ti OR ‘smart phone’:ab,ti OR ‘tablet’:ab,ti OR ‘blog’:ab,ti OR ‘blogging’:ab,ti OR ‘SMS’:ab,ti OR ‘short message servic*’:ab,ti OR ‘text message’:ab,ti OR ‘Texting e-mail’:ab,ti OR ‘email’:ab,ti OR ‘computer’:ab,ti OR ‘tablet’:ab,ti OR ‘video conferenc*’:ab,ti OR ‘video meeting’:ab,ti OR ‘interactive video’:ab,ti OR ‘TV meeting’:ab,ti OR ‘serious gam*’:ab,ti OR ‘video game*’:ab,ti OR ‘videogame’:ab,ti OR ‘gamification’:ab,ti OR ‘digital game’:ab,ti OR ‘gaming’:ab,ti OR ‘social media’:ab,ti OR ‘socialmedia’:ab,ti OR ‘social network’:ab,ti OR ‘virtual reality’:ab,ti OR ‘VR’:ab,ti OR ‘personal digital assistant’:ab,ti  #11 #4 OR #5 OR #6 OR #7 OR #8 OR #9 OR #10  #12 'health behavior'/exp  #13 ‘risk reduction behavior’:ab,ti OR ‘health behavio*’:ab,ti OR ‘health-related behavio*’:ab,ti OR ‘health related behavio*’:ab,ti OR ‘healthy lifestyle*’:ab,ti OR ‘health promoting behavio*’:ab,ti OR ‘risk reduction behavio*’:ab,ti OR ‘risk behavio*’:ab,ti OR ‘risky health’:ab,ti  #14 #12 OR #13  #15 'randomized controlled trial'/exp  #16 ‘controlled clinical trial’:ab,ti OR ‘randomized’:ab,ti OR ‘randoml’:ab,ti OR ‘trial’:ab,ti  #17 #15 OR #16  #18 ‘care-as-usual’:ab,ti OR ‘control’:ab,ti OR ‘no treatment’:ab,ti OR ‘waitlist’:ab,ti OR ‘wait-list’:ab,ti OR ‘treatment-as-usual’:ab,ti OR ‘paper’:ab,ti OR ‘active control’:ab,ti  #19 #3 AND #11 AND #14 AND #17 AND #18  #20 ‘Review’:ti OR ‘Meta’:ti OR ‘protocol’:ti  #21 #19 NOT #20 | 3091 |
| Web of Science | #1 AB=(aged OR elderly OR older adults OR aging OR veteran* OR geriatric*)  #2 AB=(Internet-Based Intervention OR Telemedicine OR Telerehabilitation OR ehealth OR e-health OR m-health OR mhealth OR mobile health OR mobile communication OR remote consult OR telecommunication* OR telehealth OR tele health OR telemedicine OR tele medicine OR telecare OR tele-care OR telehomecare OR telehealth OR tele-medicine OR tele-homecare OR telerehabilitation OR tele-rehabilitation OR tele-health OR tele-monitor OR telemonitor OR tele-management OR telemanagement OR eletronic health OR digital health OR online health OR on-line health OR computer OR internet based OR internet OR internet-based OR web OR web based OR web-based OR webcast* OR mobile technology OR mobile device OR health app* OR app* OR application* OR health applications OR phone OR cellphone OR mobile phone OR mobilephone OR smartphone OR smart phone OR tablet OR blog OR blogging OR SMS OR short message servic* OR text message OR texting e-mail OR email OR computer OR tablet OR video conferenc* OR video meeting OR interactive video OR TV meeting OR serious gam* OR video game* OR videogame OR gamification OR digital game OR gaming OR social media OR socialmedia OR social network OR virtual reality OR VR OR personal digital assistant)  #3 AB=(Health Risk Behavior OR Health Behavior OR Risk Reduction Behavior OR health behavio* OR health-related behavio* OR health related behavio* OR healthy lifestyle* OR health promoting behavio* OR risk reduction behavio* OR risk behavio* OR risky health)  #4 AB=(Randomized Controlled Trial OR controlled clinical trial OR randomized OR randoml OR trial)  #5 AB=(care-as-usual OR control OR no treatment OR waitlist OR wait-list OR treatment-as-usual OR paper OR active control)  #6 #1 AND #2 AND #3 AND #4 AND #5  #7 TI=(Review OR Meta OR protocol)  #8 #6 NOT #7 | 4675 |
| Cochrane | #1 MeSH descriptor: [Aged] in all MeSH products  #2 (elder*):ab,ti,kw OR (old*):ab,ti,kw OR (senior*):ab,ti,kw OR (veteran*):ab,ti,kw OR ( geriatric*):ab,ti,kw OR (retire*):ab,ti,kw  #3 #1 OR #2  #4 MeSH descriptor: [Internet-Based Intervention] explode all trees  #5 MeSH descriptor:[Telemedicine] explode all trees  #6 MeSH descriptor:[Telerehabilitation] explode all trees  #7 (ehealth):ab,ti,kw OR (e-health):ab,ti,kw OR (m-health):ab,ti,kw OR (mhealth):ab,ti,kw OR (mobile health):ab,ti,kw OR (mobile communication):ab,ti,kw OR (telehealth):ab,ti,kw OR (tele health):ab,ti,kw OR (tele-health):ab,ti,kw OR (telecare):ab,ti,kw OR (tele-care):ab,ti,kw OR (telehomecare):ab,ti,kw OR (tele-homecare):ab,ti,kw OR (tele-monitor):ab,ti,kw OR (telemonitor):ab,ti,kw OR (tele-management):ab,ti,kw OR (telemanagement):ab,ti,kw OR (eletronic health):ab,ti,kw OR (digital health):ab,ti,kw OR (online health):ab,ti,kw OR (on-line health):ab,ti,kw  #8 (computer):ab,ti,kw OR (internet based):ab,ti,kw OR (internet):ab,ti,kw OR (internet-based):ab,ti,kw OR (web):ab,ti,kw OR (web based):ab,ti,kw OR (web-based):ab,ti,kw OR (webcast*):ab,ti,kw OR (mobile technology):ab,ti,kw OR (mobile device):ab,ti,kw OR (health app*):ab,ti,kw OR (app*):ab,ti,kw OR (application*):ab,ti,kw OR (health applications):ab,ti,kw OR (phone):ab,ti,kw OR (cellphone):ab,ti,kw OR (mobile phone):ab,ti,kw OR (mobilephone):ab,ti,kw OR (smartphone):ab,ti,kw OR (smart phone):ab,ti,kw OR (tablet):ab,ti,kw OR (blog):ab,ti,kw OR (blogging):ab,ti,kw OR (SMS):ab,ti,kw OR (short message servic*):ab,ti,kw OR (text message):ab,ti,kw OR (texting e-mail):ab,ti,kw OR (email):ab,ti,kw OR (computer):ab,ti,kw OR (tablet):ab,ti,kw OR (video conferenc*):ab,ti,kw OR (video meeting):ab,ti,kw OR (interactive video):ab,ti,kw OR (TV meeting):ab,ti,kw OR (serious gam*):ab,ti,kw OR (video game*):ab,ti,kw OR (videogame):ab,ti,kw OR (gamification):ab,ti,kw OR (digital game):ab,ti,kw OR (gaming):ab,ti,kw OR (social media):ab,ti,kw OR (socialmedia):ab,ti,kw OR (social network):ab,ti,kw OR (virtual reality):ab,ti,kw OR (VR):ab,ti,kw OR (personal digital assistant):ab,ti,kw  #9 #4 OR #5 OR #6 OR #7 OR #8  #10 MeSH descriptor:[Health Behavior] explode all trees  #11 MeSH descriptor:[Health Risk Behaviors] explode all trees  #12 (risk reduction behavior):ab,ti,kw OR (health behavio*):ab,ti,kw OR (health-related behavio*):ab,ti,kw OR (health related behavio*):ab,ti,kw OR (healthy lifestyle*):ab,ti,kw OR (health promoting behavio*):ab,ti,kw OR (risk reduction behavio*):ab,ti,kw OR (risk behavio*):ab,ti,kw OR (risky health):ab,ti,kw  #13 #10 OR #11 OR #12  #14 MeSH descriptor:[randomized controlled trial]explode all trees  #15 (controlled clinical trial):ab,ti,kw OR (randomized):ab,ti,kw OR (randoml):ab,ti,kw OR (trial):ab,ti,kw  #16 #15 OR #14  #17 (care-as-usual):ab,ti,kw OR (control):ab,ti,kw OR (no treatment):ab,ti,kw OR (waitlist):ab,ti,kw OR (wait-list):ab,ti,kw OR (treatment-as-usual):ab,ti,kw OR (paper):ab,ti,kw OR (active control):ab,ti,kw  #18 #3 AND #9 AND #13 AND #16 AND #17  #19 (Review):ti OR (Meta):ti OR (protocol):ti  #20 #18 NOT #19 | 8190 |
| Scopus | 1 “aged” OR “elderly” OR “older adults” OR “aging” OR “veteran*” OR “geriatric*”  2 “Internet-Based Intervention” OR “Telemedicine” OR “Telerehabilitation” OR “ehealth” OR “e-health” OR “m-health” OR “mhealth” OR “mobile health” OR “mobile communication” OR “remote consult” OR “telecommunication*” OR “telehealth” OR “tele health” OR “telemedicine” OR “tele medicine” OR “telecare” OR “tele-care” OR “telehomecare” OR “telehealth” OR “tele-medicine” OR “tele-homecare” OR “telerehabilitation” OR “tele-rehabilitation” OR “tele-health” OR “tele-monitor” OR “telemonitor” OR “tele-management” OR “telemanagement” OR “eletronic health” OR “digital health” OR “online health” OR “on-line health” OR “computer” OR “internet based” OR “internet” OR “internet-based” OR “web” OR “web based” OR “web-based” OR “webcast*” OR “mobile technology” OR “mobile device” OR “health app*” OR “app*” OR “application*” OR “health applications” OR “phone” OR “cellphone” OR “mobile phone” OR “mobilephone” OR “smartphone” OR “smart phone” OR “tablet” OR “blog” OR “blogging” OR “SMS” OR “short message servic*” OR “text message” OR “texting e-mail” OR “email” OR “computer” OR “tablet” OR “video conferenc*” OR “video meeting” OR “interactive video” OR “TV meeting” OR “serious gam*” OR “video game*” OR “videogame” OR “gamification” OR “digital game” OR “gaming” OR “social media” OR “socialmedia” OR “social network” OR “virtual reality” OR “VR” OR “personal digital assistant”  3 “Health Risk Behavior” OR “Health Behavior” OR “Risk Reduction Behavior” OR “health behavio*” OR “health-related behavio*” OR “health related behavio*” OR “healthy lifestyle*” OR “health promoting behavio*” OR “risk reduction behavio*” OR “risk behavio*” OR “risky health”  4 “Randomized Controlled Trial” OR “controlled clinical trial” OR “randomized” OR “randoml” OR “trial”  5 “care-as-usual” OR “control” OR “no treatment” OR “waitlist” OR “wait-list” OR “treatment-as-usual” OR “paper” OR “active control”  5 1 AND 2 AND 3 AND 4 AND 5  6 “Review” OR “Meta”OR “protocol”  7 5 AND NOT 6 | 2021 |
| SPORTDiscus | #1 AB (aged OR elderly OR older adults OR aging OR veteran* OR geriatric*)  #2 AB (Internet-Based Intervention OR Telemedicine OR Telerehabilitation OR ehealth OR e-health OR m-health OR mhealth OR mobile health OR mobile communication OR remote consult OR telecommunication* OR telehealth OR tele health OR telemedicine OR tele medicine OR telecare OR tele-care OR telehomecare OR telehealth OR tele-medicine OR tele-homecare OR telerehabilitation OR tele-rehabilitation OR tele-health OR tele-monitor OR telemonitor OR tele-management OR telemanagement OR eletronic health OR digital health OR online health OR on-line health OR computer OR internet based OR internet OR internet-based OR web OR web based OR web-based OR webcast* OR mobile technology OR mobile device OR health app* OR app* OR application* OR health applications OR phone OR cellphone OR mobile phone OR mobilephone OR smartphone OR smart phone OR tablet OR blog OR blogging OR SMS OR short message servic* OR text message OR texting e-mail OR email OR computer OR tablet OR video conferenc* OR video meeting OR interactive video OR TV meeting OR serious gam* OR video game* OR videogame OR gamification OR digital game OR gaming OR social media OR socialmedia OR social network OR virtual reality OR VR OR personal digital assistant)  #3 AB (Health Risk Behavior OR Health Behavior OR Risk Reduction Behavior OR health behavio* OR health-related behavio* OR health related behavio* OR healthy lifestyle* OR health promoting behavio* OR risk reduction behavio* OR risk behavio* OR risky health)  #4 AB (Randomized Controlled Trial OR controlled clinical trial OR randomized OR randoml OR trial)  #5 AB (care-as-usual OR control OR no treatment OR waitlist OR wait-list OR treatment-as-usual OR paper OR active control)  #6 #1 AND #2 AND #3 AND #4 AND #5  #7 TI=(Review OR Meta OR protocol)  #8 #6 NOT #7 | 86 |

All=1010+3091+4675+8190+2021+86=19072
