# Supplementary material for: Assessing the Effectiveness of eHealth Interventions to Manage Multiple Lifestyle Risk Behaviors Among Older Adults: Systematic Review and Meta-Analysis
Source: J Med Internet Res. 2024 Jul 31;26:e58174. doi: 10.2196/58174 (PMC11325121; doi:10.2196/58174)
Supplement: Multimedia Appendix 3 [file jmir_v26i1e58174_app3.docx]

**Multimedia Appendix 3: The measurement tools for different outcomes**

| **Health behaviors** | **Measurement tools of interests** |
| --- | --- |
| PA | the International Physical Activity Questionnaire (IPAQ); the short form IPAQ; the Global Physical Activity Questionnaire (GPAQ); the validated Recent Physical Activity Questionnaire (RPAQ); the Godin Exercise Questionnaire; BodyMedia SenseWear ‘Core’ monitors; an interview-administered physical activity questionnaire; Pedometers; the Short Questionnaire to Assess Health Enhancing Physical Activity (SQUASH); the Godin Leisure-Time Exercise Questionnaire; accelerometer; Actiwatch; the Physical Activity Scale for the Elderly (PASE); the CHAMPS instrument; the 7-Day Physical Activity Recall; the Women’s Health Initiative questionnaire; the Community Health Activities Model Program for Seniors questionnaire |
| Diet | three months recalls; 24-hour recall method; the Block Food Frequency Questionnaire; three-Day Food Records; the food frequency questionnaire (FFQ); the Dutch Standard Questionnaire on Food Consumption; the Thompson Food Frequency Questionnaire |
| SB | BodyMedia SenseWear ‘Core’ monitors; accelerometer |
| Sleep | the 5-item validated Women’s Health Initiative Insomnia Rating Scale (WHIIRS); accelerometer |
