# Supplementary figures and images for: Assessing the Effectiveness of eHealth Interventions to Manage Multiple Lifestyle Risk Behaviors Among Older Adults: Systematic Review and Meta-Analysis
Source: J Med Internet Res. 2024 Jul 31;26:e58174. doi: 10.2196/58174 (PMC11325121; doi:10.2196/58174)

**Multimedia Appendix 4: Risk of bias**

**Risk of Bias Summary**


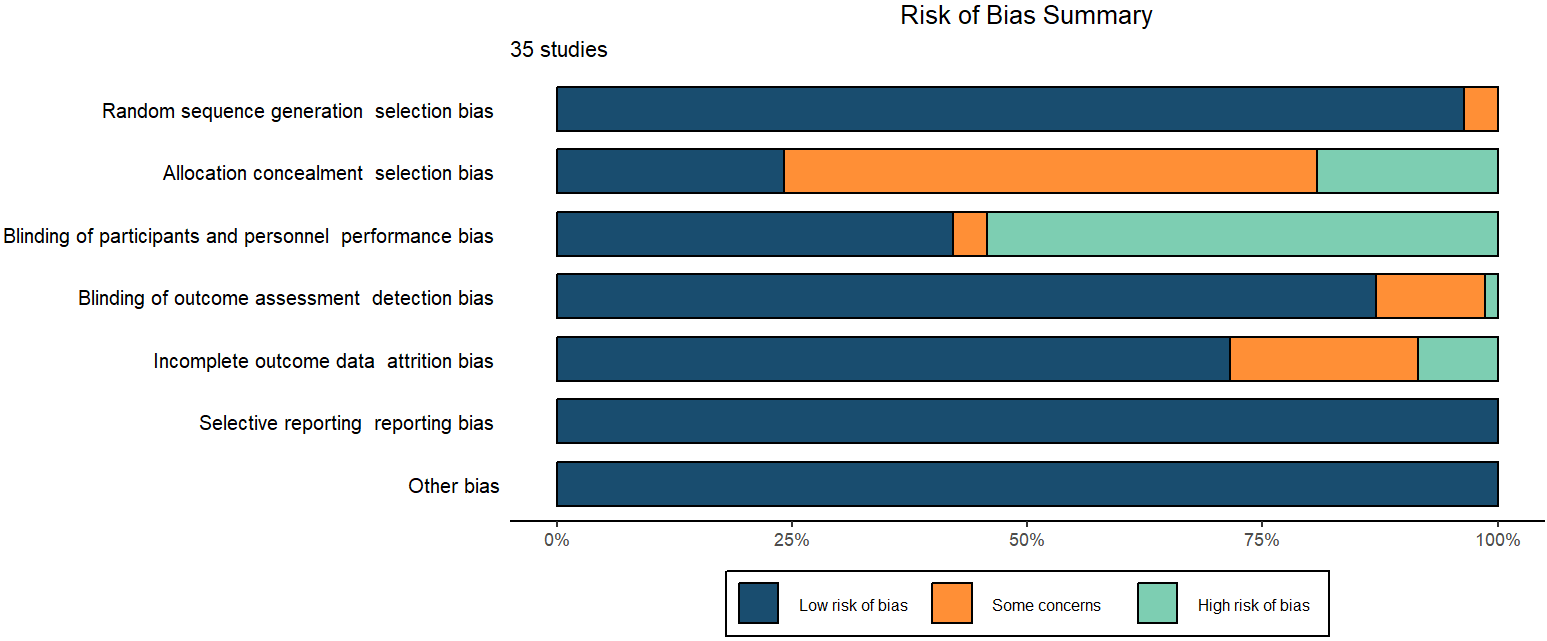


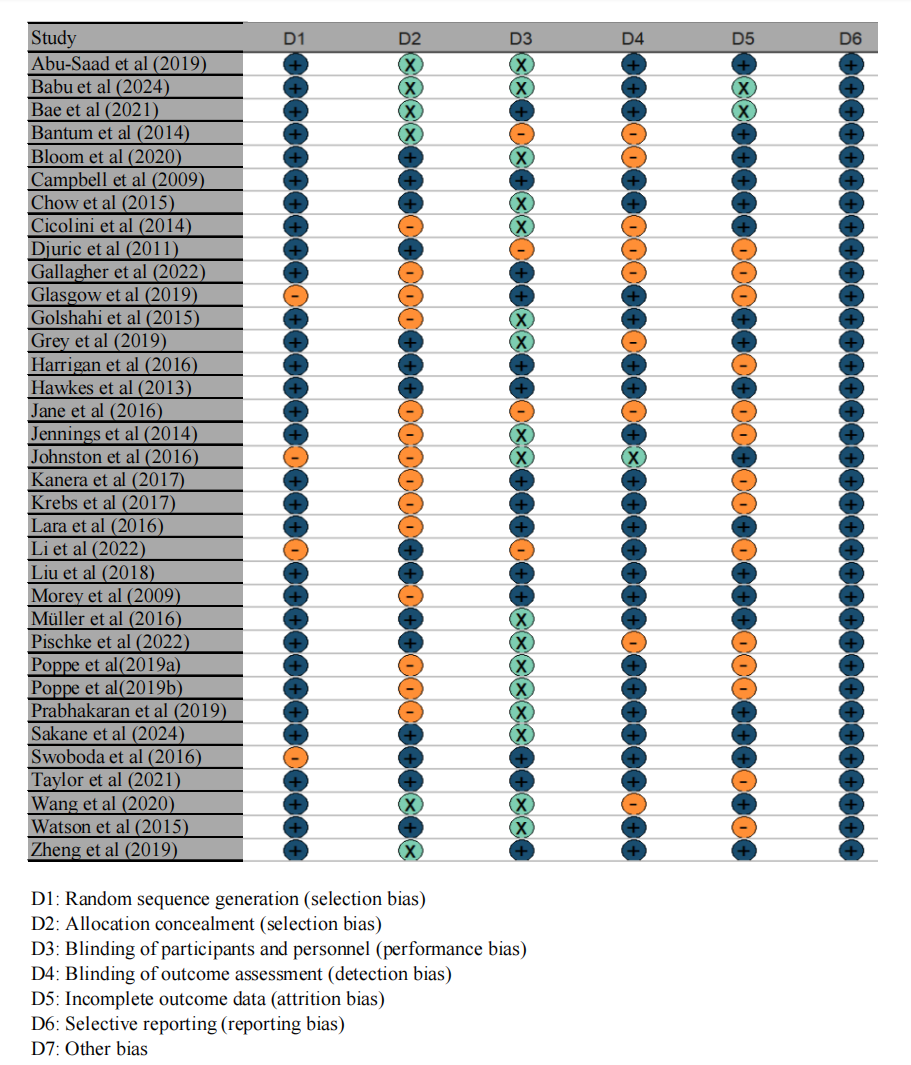

Supplement: Multimedia Appendix 4 [file jmir_v26i1e58174_app4.docx]
