# Supplementary material for: Assessing the Effectiveness of eHealth Interventions to Manage Multiple Lifestyle Risk Behaviors Among Older Adults: Systematic Review and Meta-Analysis
Source: J Med Internet Res. 2024 Jul 31;26:e58174. doi: 10.2196/58174 (PMC11325121; doi:10.2196/58174)
Supplement: Multimedia Appendix 5 [file jmir_v26i1e58174_app5.docx]

**Multimedia Appendix 5: GRADE (Grading of Recommendations Assessment, Development, and Evaluation) assessment results.**

| **Summary of findings table for the effectiveness of eHealth interventions compared to education as usual and/or non-eHealth interventions in older adults to prevent behavioral risk factors** | | | | | | |
| --- | --- | --- | --- | --- | --- | --- |
| **Patient or population:** older adults ≥50 **Settings:** no **Intervention:** e-health intervention **Comparison:** waitlist, care as usual, or active control | | | | | | |
| **Outcomes** | **Illustrative comparative risks* (95% CI)** | | **Relative effect (95% CI)** | **No of Participants (studies)** | **Quality of the evidence (GRADE)** | **Comments** |
|  | Assumed risk | Corresponding risk |  |  |  |  |
|  | **Control** | **E-health** |  |  |  |  |
| **smoking** | **Study population** | | **OR 2.09**  (1.62 to 2.70) | 1731 (10 studies) | ⊕⊝⊝⊝ **very low**^1,2^ |  |
|  | **258 per 1000** | **420 per 1000** (360 to 484) |  |  |  |  |
|  | **Moderate** | |  |  |  |  |
|  | **157 per 1000** | **284 per 1000** (234 to 335) |  |  |  |  |
| **alcohol use** | **Study population** | | **OR 0.75**  (0.26 to 2.18) | 380 (4 studies) | ⊕⊝⊝⊝ **very low**^1,3^ |  |
|  | **555 per 1000** | **483 per 1000** (245 to 731) |  |  |  |  |
|  | **Moderate** | |  |  |  |  |
|  | **400 per 1000** | **333 per 1000** (148 to 592) |  |  |  |  |
| **regular exercise** | **Study population** | | **OR 4.03**  (3.14 to 5.18) | 1967 (8 studies) | ⊕⊕⊕⊕ **high** |  |
|  | **148 per 1000** | **413 per 1000** (354 to 474) |  |  |  |  |
|  | **Moderate** | |  |  |  |  |
|  | **107 per 1000** | **326 per 1000** (273 to 383) |  |  |  |  |
| **Inactive** | **Study population** | | **OR 4.51**  (3.25 to 6.26) | 1443 (4 studies) | ⊕⊕⊕⊕ **high** |  |
|  | **169 per 1000** | **479 per 1000** (399 to 561) |  |  |  |  |
|  | **Moderate** | |  |  |  |  |
|  | **116 per 1000** | **372 per 1000** (299 to 451) |  |  |  |  |
| **Sleep** |  | The mean sleep in the intervention groups was **0.12 standard deviations higher** (0.04 lower to 0.29 higher) |  | 558 (3 studies) | ⊕⊕⊝⊝ **low**^3^ | SMD 0.12 (-0.04 to 0.29) |
| **Fruits** |  | The mean fruits in the intervention groups was **0.18 standard deviations higher** (0.04 to 0.32 higher) |  | 758 (8 studies) | ⊕⊕⊝⊝ **low**^3^ | SMD 0.18 (0.04 to 0.32) |
| **vegetables** |  | The mean vegetables in the intervention groups was **0.17 standard deviations higher** (0.05 to 0.28 higher) |  | 1251 (10 studies) | ⊕⊕⊕⊕ **high** | SMD 0.17 (0.05 to 0.28) |
| **Fruits and vegetables** |  | The mean fruits and vegetables in the intervention groups was **0.33 standard deviations higher** (0.06 to 0.59 higher) |  | 1374 (7 studies) | ⊕⊕⊕⊕ **high** | SMD 0.33 (0.06 to 0.59) |
| **fat** |  | The mean fat in the intervention groups was **0.23 standard deviations lower** (0.33 to 0.13 lower) |  | 1599 (9 studies) | ⊕⊕⊕⊕ **high** | SMD -0.23 (-0.33 to -0.13) |
| **sugar** |  | The mean sugar in the intervention groups was **0.11 standard deviations lower** (0.36 lower to 0.15 higher) |  | 241 (5 studies) | ⊕⊕⊕⊝ **moderate**^4^ | SMD -0.11 (-0.36 to 0.15) |
| **fiber** |  | The mean fiber in the intervention groups was **0.04 standard deviations higher** (0.12 lower to 0.2 higher) |  | 620 (7 studies) | ⊕⊕⊝⊝ **low**^3^ | SMD 0.04 (-0.12 to 0.2) |
| **Energy intake** |  | The mean energy intake in the intervention groups was **0.28 standard deviations lower** (0.55 to 0.01 lower) |  | 213 (5 studies) | ⊕⊕⊕⊝ **moderate**^4^ | SMD -0.28 (-0.55 to -0.01) |
| **Self-reported total PA** |  | The mean self-reported pa in the intervention groups was **0.22 standard deviations higher** (0.02 lower to 0.43 higher) |  | 2689 (17 studies) | ⊕⊝⊝⊝ **very low**^1,2,5^ | SMD 0.22 (0.02 to 0.43) |
| **Objectively total measured PA** |  | The mean objectively measured pa in the intervention groups was **0.09 standard deviations higher** (0.23 lower to 0.42 higher) |  | 153 (5 studies) | ⊕⊝⊝⊝ **very low**^1,3^ | SMD 0.09 (-0.23 to 0.42) |
| **Self-reported MVPA** |  | The mean self-reported mvpa in the intervention groups was **0.1 standard deviations lower** (0.57 lower to 0.36 higher) |  | 1510 (11 studies) | ⊕⊝⊝⊝ **very low**^1,5^ | SMD -0.1 (-0.57 to 0.36) |
| **Objectively measured MVPA** |  | The mean objectively measured mvpa in the intervention groups was **0.25 standard deviations higher** (0.09 to 0.41 higher) |  | 595 (7 studies) | ⊕⊝⊝⊝ **very low**^1,2,5^ | SMD 0.25 (0.09 to 0.41) |
| **daily steps** |  | The mean daily steps in the intervention groups was **0.21 standard deviations higher** (0.01 lower to 0.44 higher) |  | 327 (7 studies) | ⊕⊝⊝⊝ **very low**^1,3^ | SMD 0.21 (-0.01 to 0.44) |
| **Self-reported SB** |  | The mean self-reported sb in the intervention groups was **0.14 standard deviations lower** (0.31 lower to 0.02 higher) |  | 589 (9 studies) | ⊕⊝⊝⊝ **very low**^1,5^ | SMD -0.14 (-0.31 to 0.02) |
| **Objectively measured SB** |  | The mean objectively measured sb in the intervention groups was **0.12 standard deviations lower** (0.3 lower to 0.07 higher) |  | 484 (7 studies) | ⊕⊝⊝⊝ **very low**^1,3^ | SMD -0.12 (-0.3 to 0.07) |
| *The basis for the **assumed risk** (e.g. the median control group risk across studies) is provided in footnotes. The **corresponding risk** (and its 95% confidence interval) is based on the assumed risk in the comparison group and the **relative effect** of the intervention (and its 95% CI). **CI:** Confidence interval; **OR:** Odds ratio; SMD: Standardised mean difference | | | | | | |
| GRADE Working Group grades of evidence **High quality:** Further research is very unlikely to change our confidence in the estimate of effect.  **Moderate quality:** Further research is likely to have an important impact on our confidence in the estimate of effect and may change the estimate. **Low quality:** Further research is very likely to have an important impact on our confidence in the estimate of effect and is likely to change the estimate. **Very low quality:** We are very uncertain about the estimate. | | | | | | |
| 1. downgraded due to high or unclear risk of bias among multiple domains  2. downgraded due to high and/or significant heterogeneity  3. Downgraded due to small sample sizes and imprecise measurements (very wide CI and/or CI including the null value)  4. downgraded due to small sample size for outcome indicators  5. downgraded due to imprecise measurement (very wide CI and/or CI including the null value) | | | | | | |
