# Supplementary material for: Assessing the Effectiveness of eHealth Interventions to Manage Multiple Lifestyle Risk Behaviors Among Older Adults: Systematic Review and Meta-Analysis
Source: J Med Internet Res. 2024 Jul 31;26:e58174. doi: 10.2196/58174 (PMC11325121; doi:10.2196/58174)
Supplement: Multimedia Appendix 6 [file jmir_v26i1e58174_app6.docx]

**Multimedia Appendix 6: Forest plots**

**Figure S1. Forest plots: eHealth intervention versus control group effect on PA.**


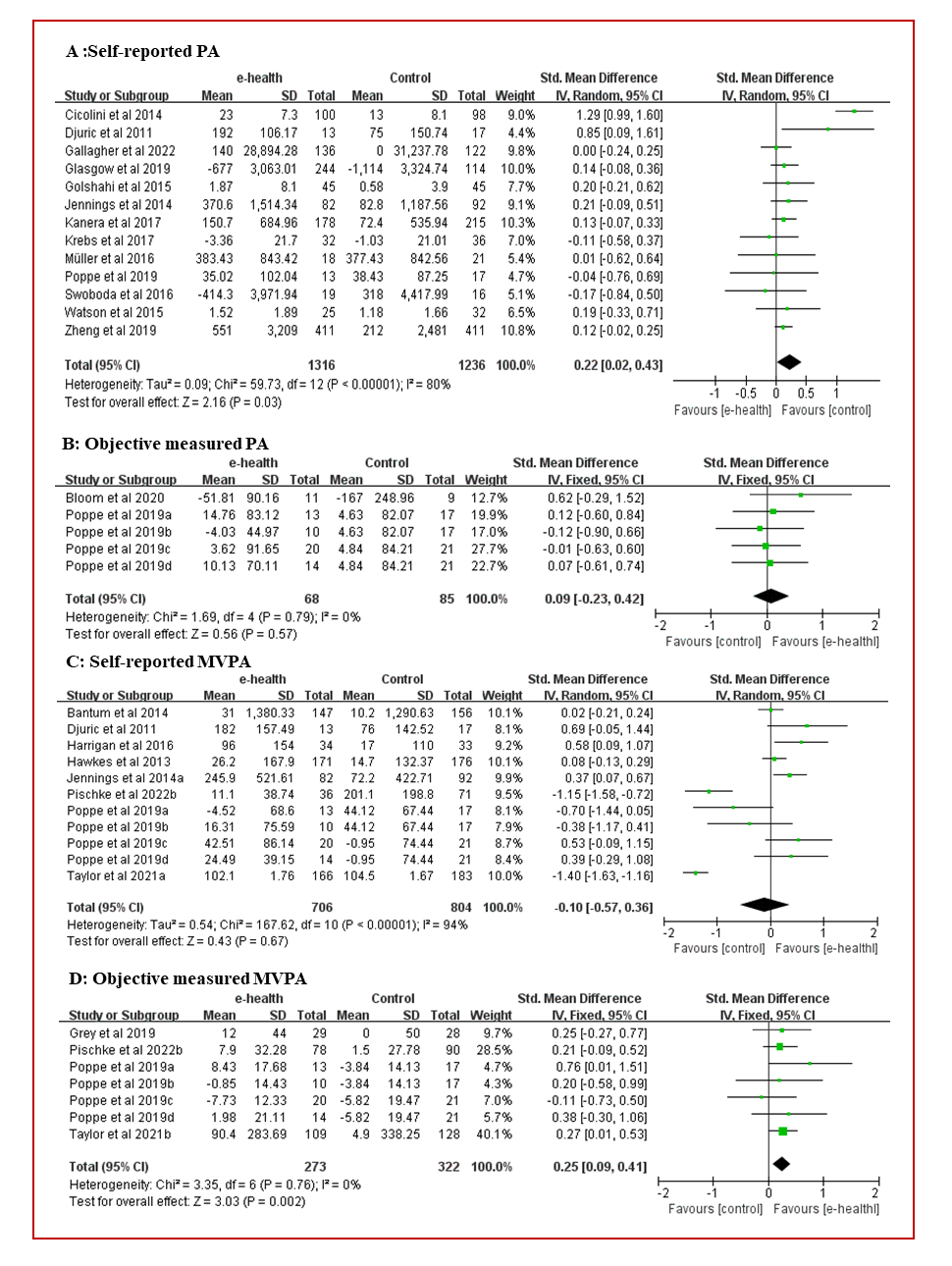


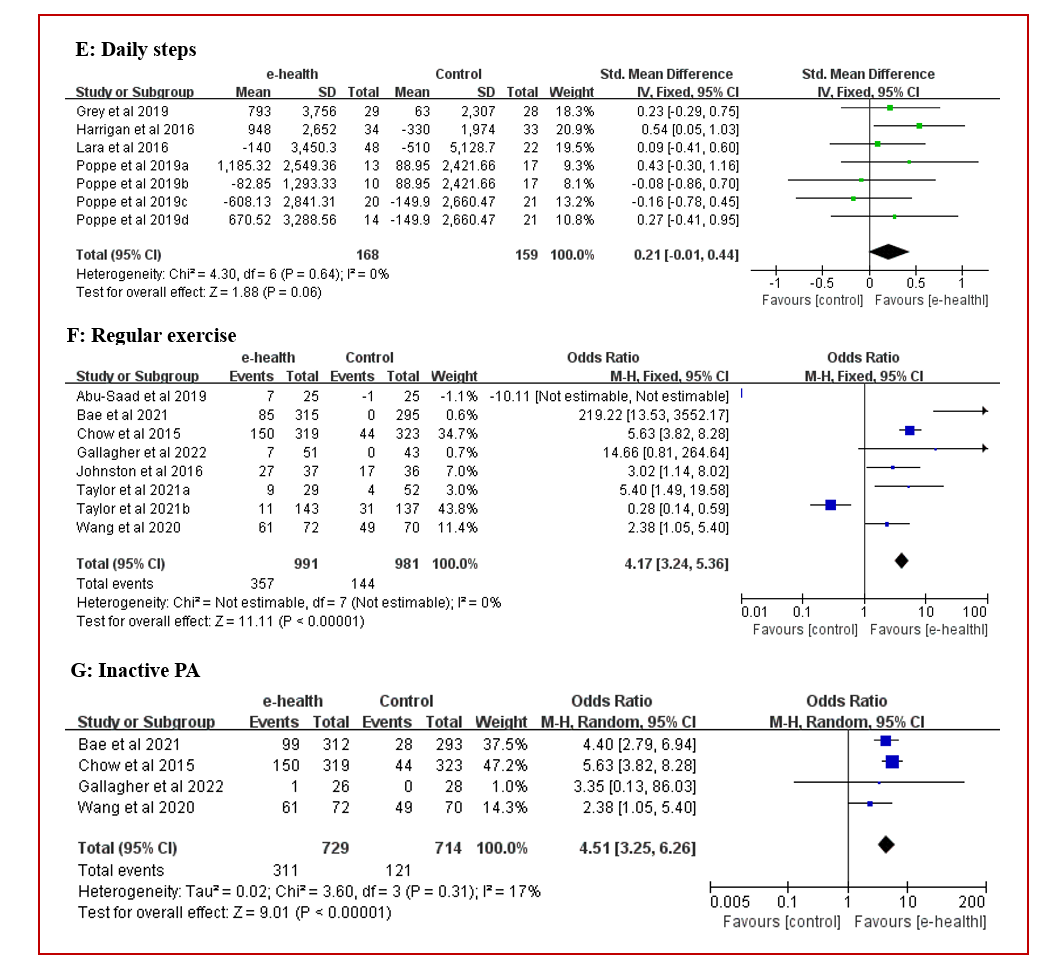


**Figure S2. Forest plots: eHealth intervention versus control group effect on SB.**

**
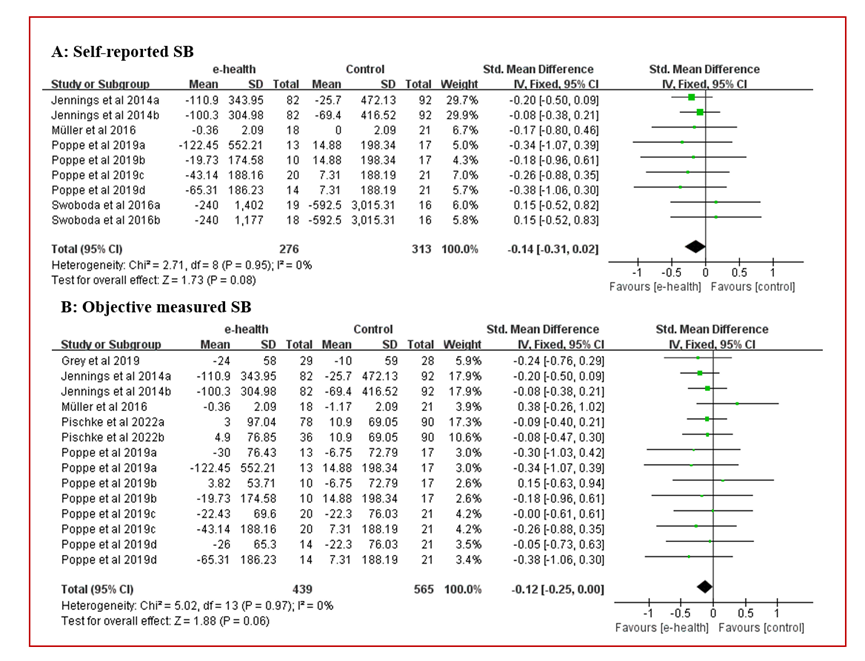
**

**Figure S3. Forest plots: eHealth intervention versus control group effect on diet.**


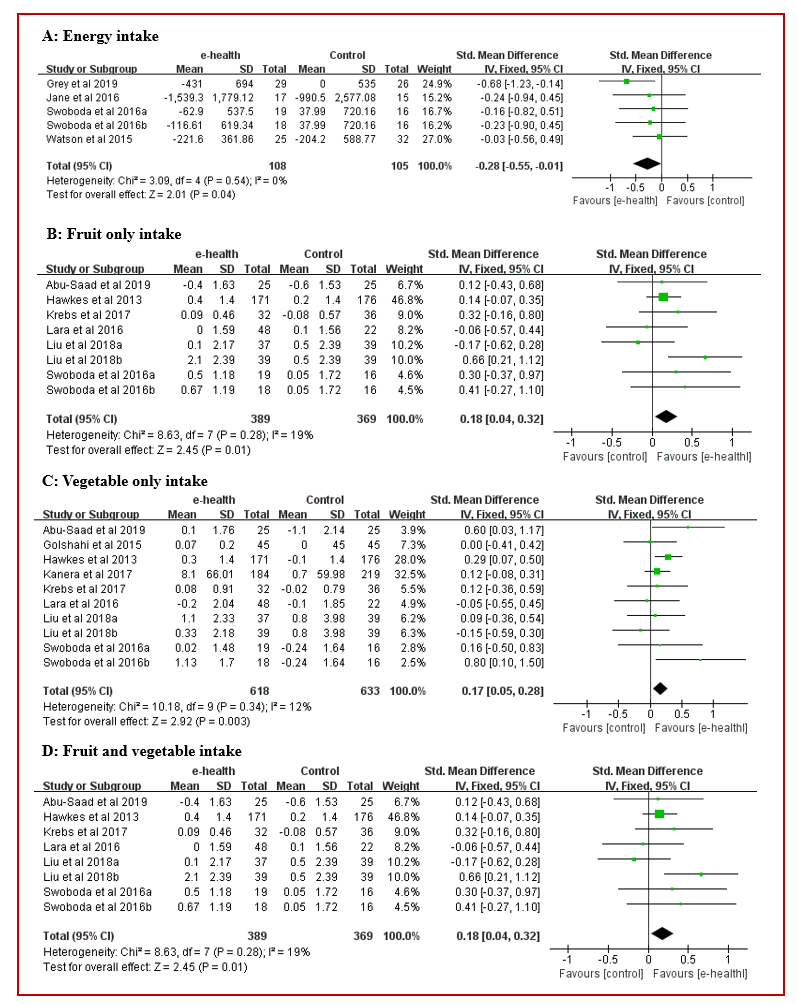


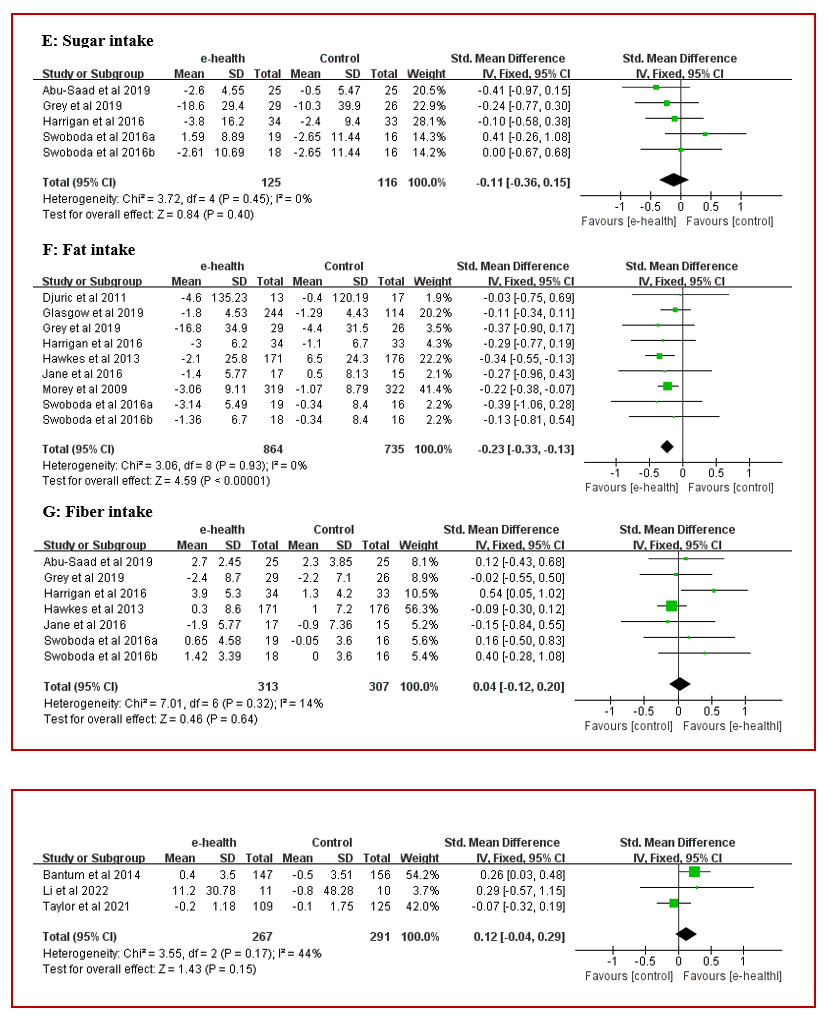


**Figure S4. Forest plots: eHealth intervention versus control group effect on smoking and alcohol use.**

**
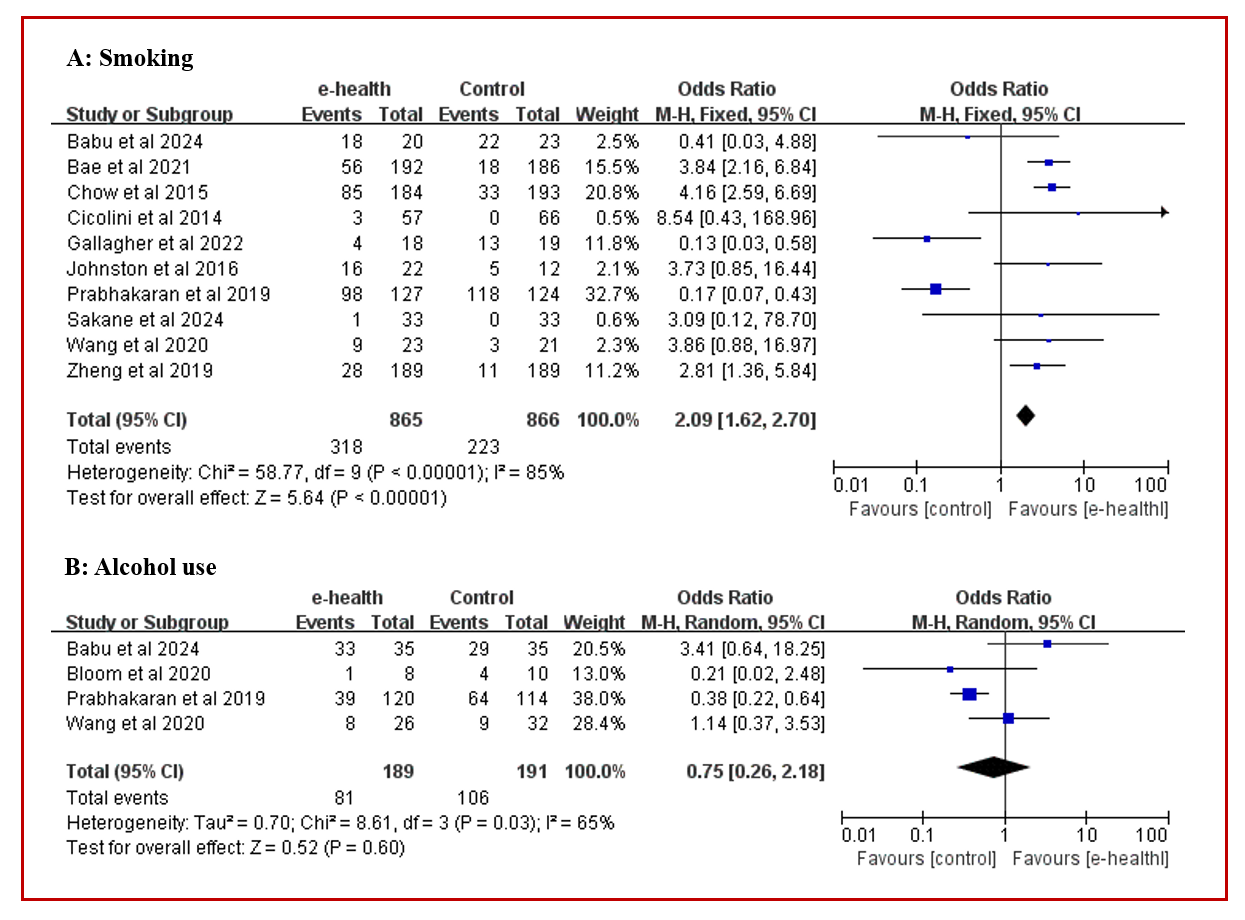
**

**Figure S5. Forest plots: eHealth intervention versus control group effect on sleep.**

**
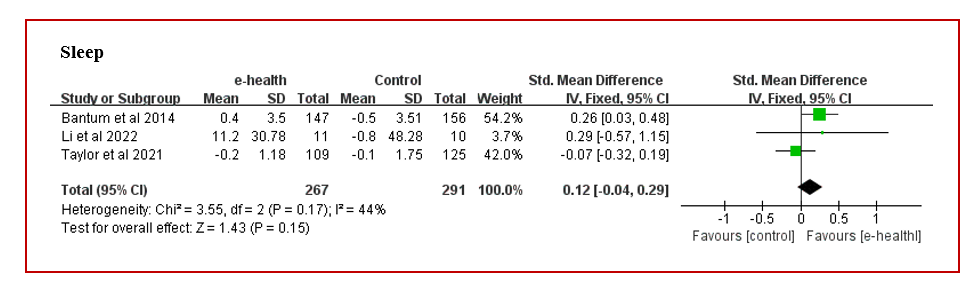
**
