# Supplementary material for: Assessing the Effectiveness of eHealth Interventions to Manage Multiple Lifestyle Risk Behaviors Among Older Adults: Systematic Review and Meta-Analysis
Source: J Med Internet Res. 2024 Jul 31;26:e58174. doi: 10.2196/58174 (PMC11325121; doi:10.2196/58174)

**Multimedia Appendix 7: Sensitive analysis.**

Forest plot: sensitivity analyses for (A): Self-reported PA; (B): Self-reported MVPA; (C) smoking; (D)Alcohol use


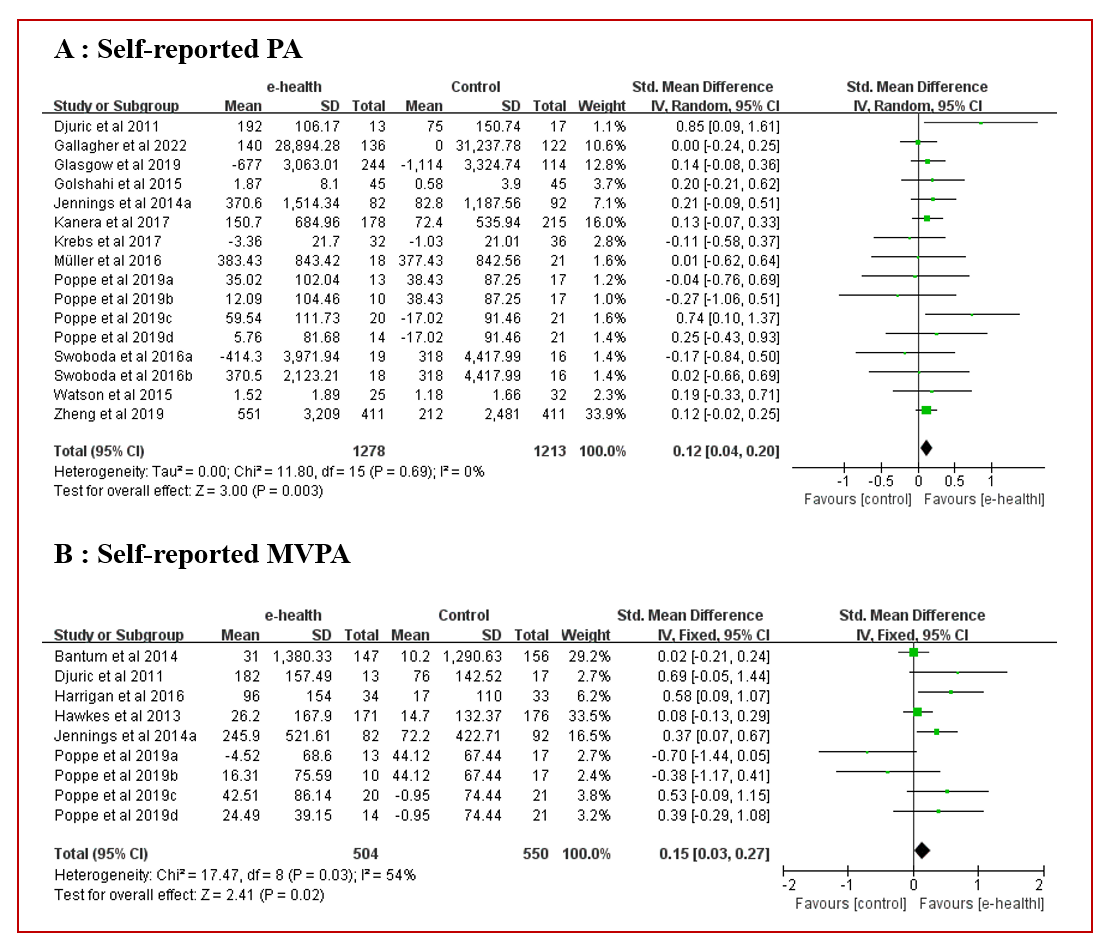


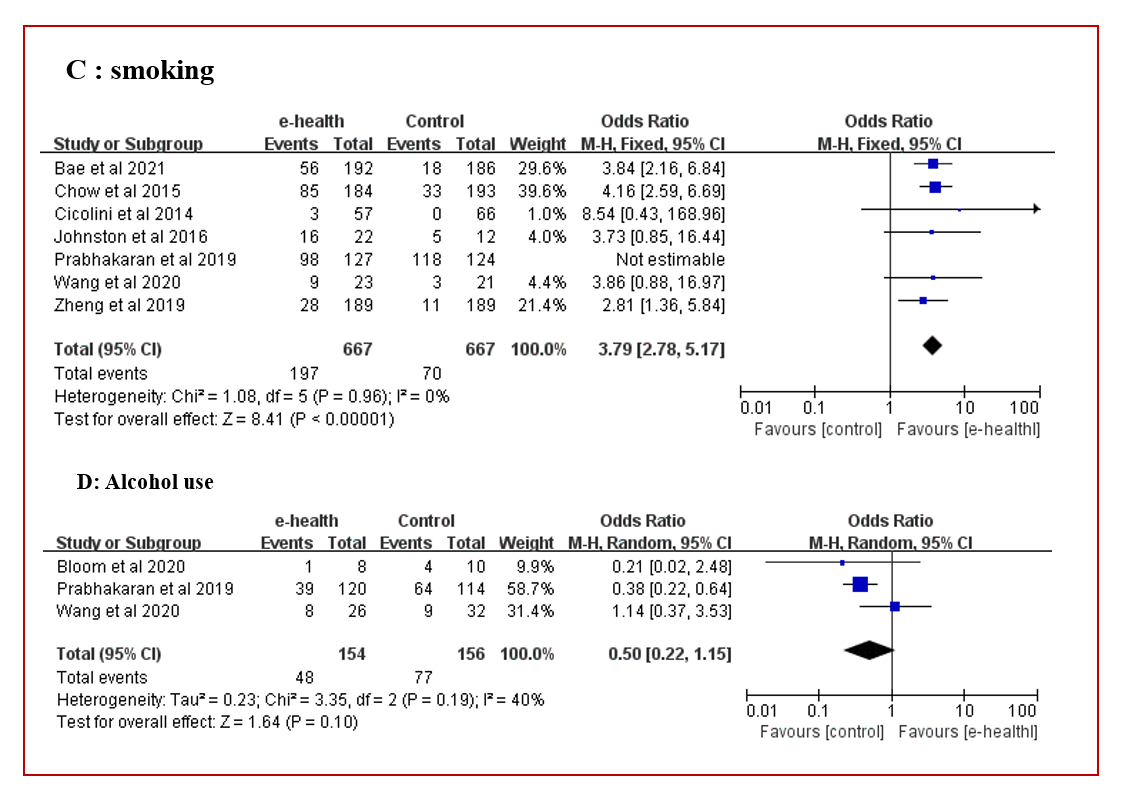

Supplement: Multimedia Appendix 7 [file jmir_v26i1e58174_app7.docx]
